# MIDA boronate allylation – synthesis of ibuprofen†

**DOI:** 10.1039/d0ra03338c

**Published:** 2020-08-18

**Authors:** David Phillips, Glen Brodie, Sarah Memarzadeh, Gi Lum Tang, David J. France

**Affiliations:** School of Chemistry, University of Glasgow Glasgow G12 8QQ UK david.france@glasgow.ac.uk

## Abstract

MIDA boronates are among the most useful reagents for the Suzuki–Miyaura reaction. This chemistry typically generates new bonds between two aromatic rings, thereby restricting access to important areas of chemical space. Here we demonstrate the coupling of MIDA boronates to allylic electrophiles, including a new synthesis of the well-known COX inhibitor ibuprofen.

## Abstract 2

Here we demonstrate the coupling of MIDA boronates to allylic electrophiles, including a new synthesis of the well-known COX inhibitor ibuprofen.

## Graphical abstract

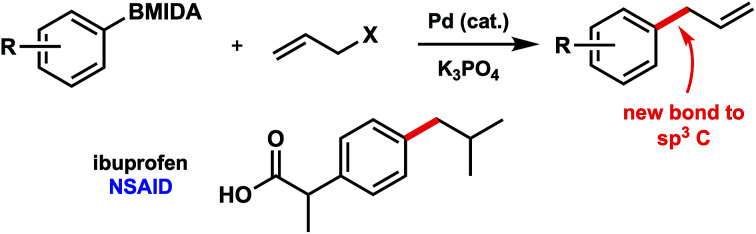

## Introduction

**Fig. 1:**
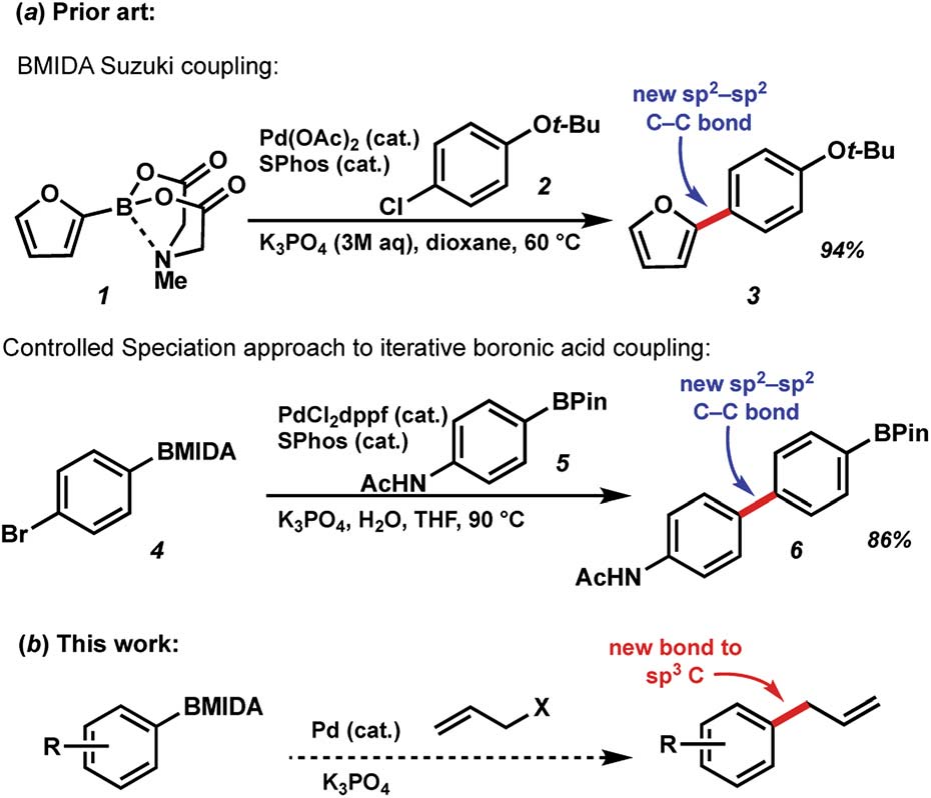
(a) Previous BMIDA coupling strategies (b) proposed BMIDA allylation to expand scope to generating new bonds to sp^3^-hybridized carbons.

*N*-Methyliminodiacetic acid (MIDA) boronates are one of the most useful organoboranes for the Suzuki–Miyaura cross-coupling strategy, as pioneered by Burke *et al.* (Fig. 1).^1^ In addition to helping to suppress undesired side reactions such as protodeboronation, these BMIDA compounds have shown promise in enabling carbon–carbon bond formation through an iterative “building block” approach. This chemistry takes advantage to two key properties of MIDA boronates to enable iterative couplings: the orthogonal reactivity of these boronates under aqueous *vs.* anhydrous conditions, and crucially a “catch-and-release” purification based on the chromatographic properties of the BMIDA functionality. By deprotecting MIDA boronates under aqueous conditions, followed by Pd-catalyzed coupling, a wide array of targets have been prepared using this methodology including natural products, pharmaceuticals, biological probes, and materials components.^2^ Related work by Watson *et al.* has used a controlled speciation approach for iterative biaryl coupling of boronic acid pinacol esters with MIDA boronates.^3^ While iterative strategies are widely used for generating biopolymers like polypeptides and polynucleic acids, the diversity of chemical bonds found in small molecules has hindered the development of an approach to preparing these compounds that is both modular and general. Iterative synthesis strategies that can address the challenging structural complexity posed by small molecules thus have the potential to transform the way synthetic chemistry is conducted.^4^

**Fig. 2:**
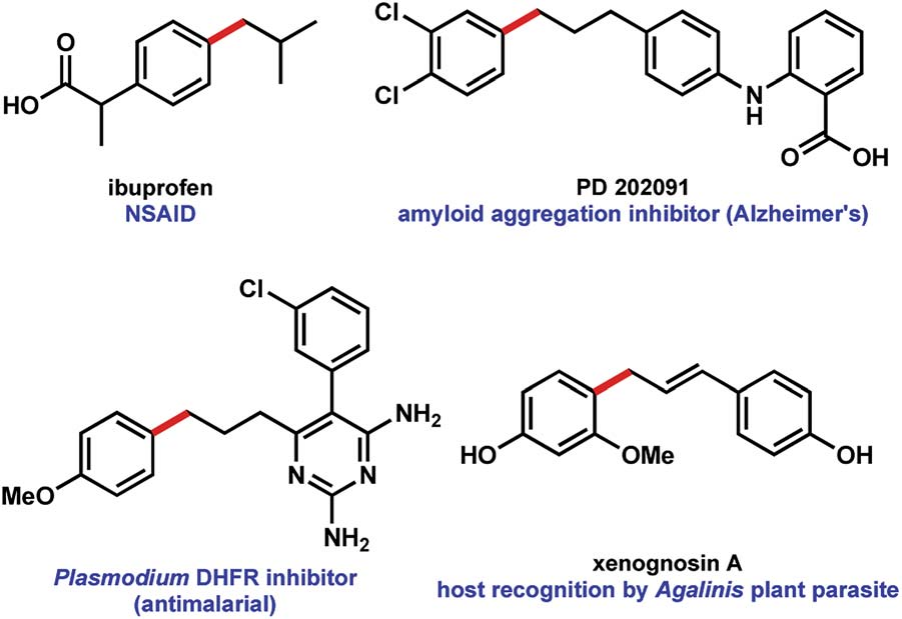
Potential bioactive targets for a MIDA boronate allylation approach to C–C coupling.^9^

Use of MIDA boronates in Pd-catalyzed coupling has enabled the synthesis of a wide array of targets.^2,4^ A hallmark of these Pd-catalyzed C–C bond formations is that they are used almost universally to couple two carbons that are sp^2^ hybridised, most commonly 2 aromatic rings. The relative ease with which flat aromatic rings can be coupled has contributed to the proliferation of these structures in MedChem programmes, however it is now deemed partially responsible for the current crisis in compound attrition rate.^5^ This drawback is also important because increased sp^3^-C content correlates to clinical success.^6^ Very few examples of coupling MIDA boronates to sp^3^ hybridised carbons are to be found in the literature,^7^ and we chose to further explore this chemistry (Fig. 1b), including in the context of drug synthesis. Our approach builds upon the well precedented allylation of boronic acids,^8^ while enabling the synthesis of new targets that could not be accessed using standard C_sp^2^_–C_sp^2^_ MIDA boronate coupling chemistry (Fig. 2).

## Results and discussion

**Fig. 3:**
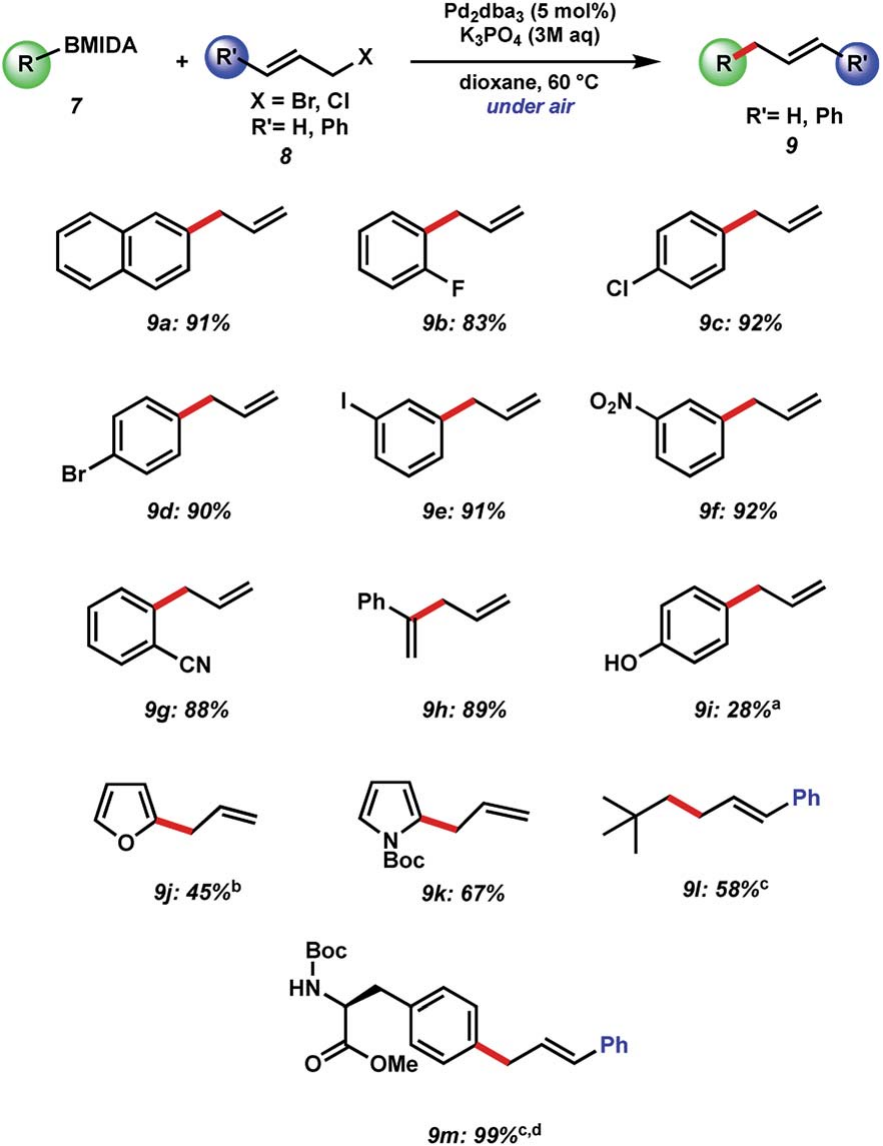
Substrate scope of BMIDA precursor with allyl bromide and cinnamyl chloride. Yields reported are by NMR unless stated. ^a^K_3_PO_4_ replaced with NaHCO_3_. ^b^Reaction carried out at room temperature. ^c^Dioxane replaced with benzene. ^d^Isolated yield.

Our initial efforts to allylate aryl BMIDA compounds were quickly rewarded, as 2-naphthyl MIDA boronate could be efficiently coupled with allyl bromide using standard hydrolysis conditions (Fig. 3). Pleasingly, the reaction could be extended to aryl halide containing substrates functionalized at each position of a phenyl ring. Clearly these functional handles allow for further couplings. Furthermore, electron withdrawing nitro and cyano substituents were well tolerated, and a vinyl MIDA boronate could also be coupled. Use of an unprotected phenol resulted in a decreased yield of the desired product of C–C bond formation, due in large part to competing *O*-allylation, even when K_3_PO_4_ was replaced with the milder base NaHCO_3_. 2-MIDA boronate substituted furan and *N*-Boc-pyrrole could also be allylated in moderate yield. Attempted allylation of other heterocycles including 3-pyridyl MIDA boronate was less successful, due in part to competing side reactions such as *N*-allylation. Interestingly, we were able to couple neo-pentyl BMIDA to cinnamyl chloride resulting in a new sp^3^–sp^3^ C–C bond (9l). This process did not prove general, with other sp^3^–sp^3^ candidates such as methyl BMIDA and cyclobutyl BMIDA having limited success (data not shown). Finally, we were able to couple a BMIDA-modified phenylalanine to cinnamyl chloride in excellent yield, demonstrating this methodology's potential use in peptide modification.

**Fig. 4:**
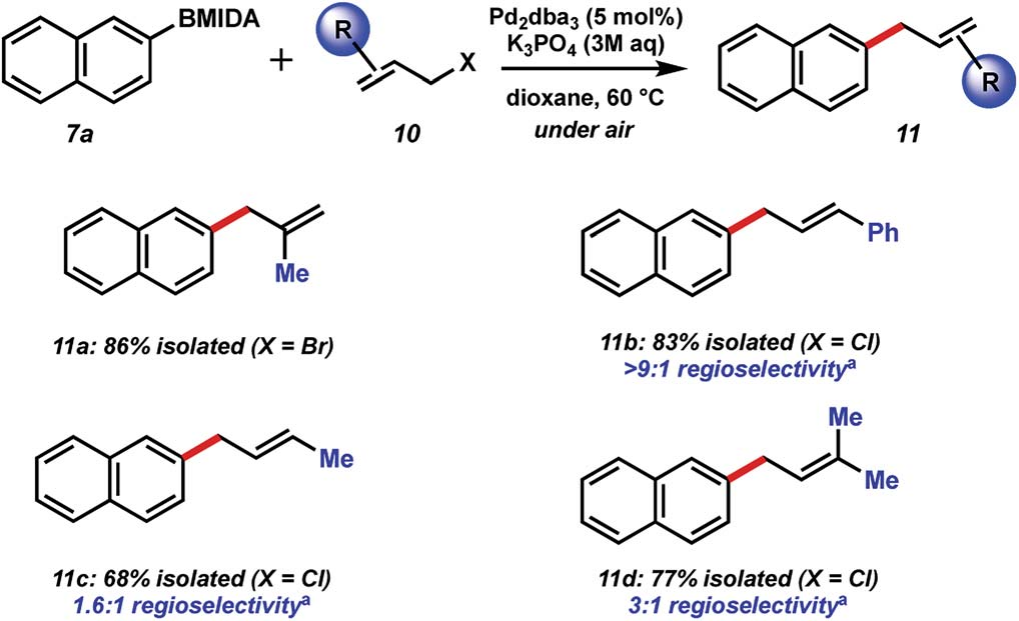
Substrate scope of allyl halide partner. ^a^Major regioisomer shown.

Next, we chose to examine the substrate tolerance on the allyl halide component by coupling 2-naphthyl MIDA boronate 7a with a range of commercial allyl halides (Fig. 4). 2-Methyl allyl bromide reacted successfully in comparable yield to the unfunctionalized system above. Coupling of cinnamyl chloride also proceeded well, and with excellent regiocontrol. BMIDA allylation with 1-methyallyl chloride and prenyl chloride resulted in decreased regioselectivity, though still good isolated yields.

**Fig. 5:**
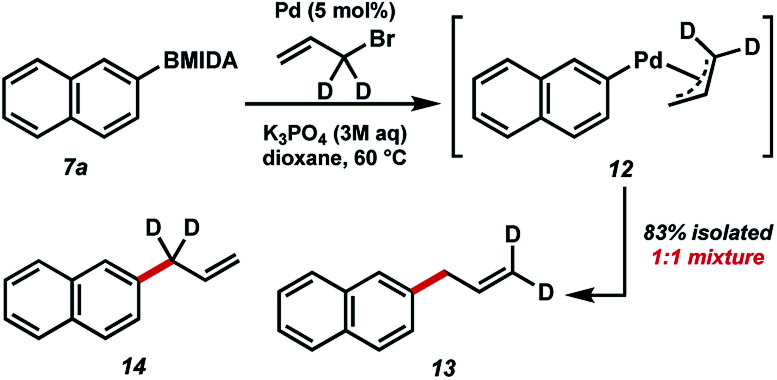
Isotopically labelled mechanistic probe.

In order to study the mechanism of the BMIDA allylation, we undertook a deuterium labelling study using dideuterioallyl bromide (Fig. 5). Coupling with 2-naphthyl MIDA boronate proceeded well, providing deuterated products 13 and 14 in 83% combined yield, but more importantly in a 1 : 1 ratio. This outcome is consistent with the intermediacy of π-allyl species 12, formed by oxidative addition to the allyl halide, and transmetallation with the boronic acid generated *in situ*.

**Fig. 6:**
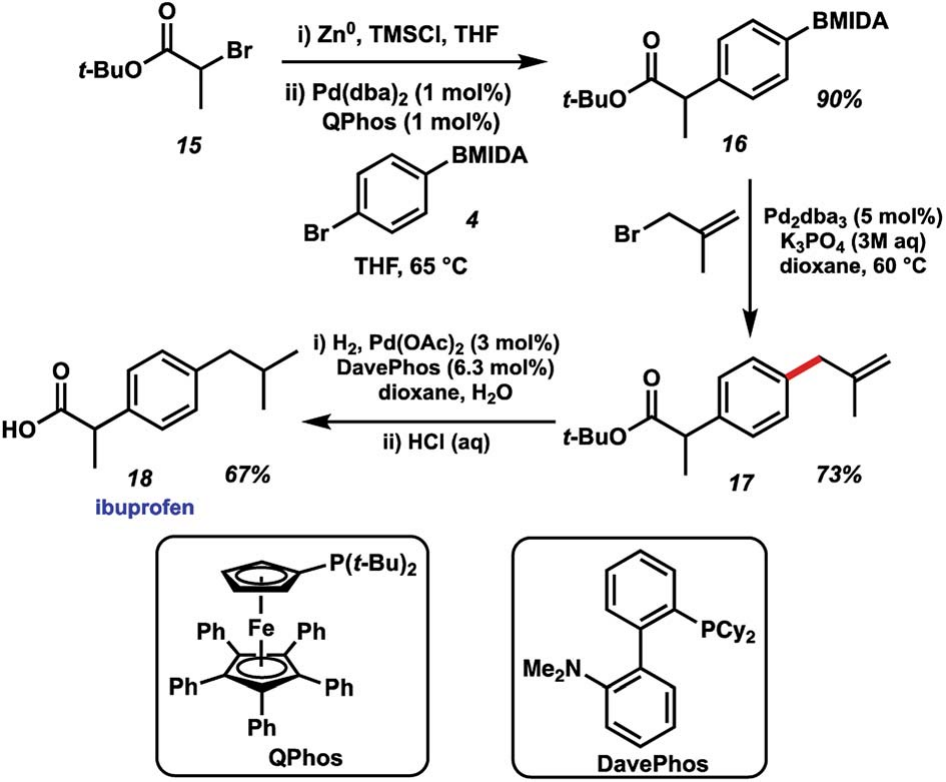
Synthesis of ibuprofen using BMIDA allylation.

Finally, we chose to exemplify the utility of the BMIDA allylation methodology by featuring it in the synthesis of the well-known NSAID ibuprofen (Fig. 6). Our initial approach called for an enolate arylation reaction of a propionate ester with *p*-bromo aryl BMIDA 4.^10^ Several conditions proved unsuccessful, likely due to complications arising from the MIDA functional group. Recourse to a Negishi arylation^11^ of α-bromo-*t*-butyl propionate however proved successful using QPhos as a ligand for Pd.^12^ Pleasingly, BMIDA allylation of 16 proceeded well, providing the coupled product in 73% isolated yield. Completion of the synthesis of ibuprofen was realized by alkene hydrogenation and deprotection of the carboxylic acid. Attempts to use the same Pd source to effect multiple transformations in this synthesis met with limited success.

## Conclusions

We have expanded the scope of MIDA boronate coupling to generate new bonds to sp^3^-hybridized carbons directly. The coupling is tolerant of a range of functional groups, including aryl halides, can be used with different allyl halide electrophiles, and is competent for sp^3^–sp^3^ C–C bond formation. This transformation can serve as a useful addition to the BMIDA-based approach to generating small molecules, as shown by a new synthesis of the widely used drug ibuprofen.

## Experimental section

Reactions involving air-sensitive agents and dry solvents were performed in glassware that had been dried in an oven (150 °C) or flame-dried prior to use. These reactions were carried out with the exclusion of air using an argon atmosphere. NMR spectra were recorded on a Bruker DPX-400 spectrometer (^1^H NMR at 400 MHz and ^13^C NMR at 100 MHz) or a Bruker DPX-500 spectrometer (^1^H NMR at 500 MHz and ^13^C NMR at 125 MHz). Chemical shifts are reported in ppm. ^1^H NMR spectra were recorded with CDCl_3_ or d_6_-DMSO as the solvent using residual proton-containing solvent as internal standard, and for ^13^C NMR spectra the chemical shifts are reported relative to the central resonance of CDCl_3_ or d_6_-DMSO. Signals in NMR spectra are described as singlet (s), doublet (d), triplet (t), multiplet (m), *etc*. or combination of these, which refers to the spin–spin coupling pattern observed. Spin–spin coupling constants reported are uncorrected. Two-dimensional (COSY, HSQC, HMBC, NOESY) NMR spectroscopy was used where appropriate to assist the assignment of signals in the ^1^H and ^13^C NMR spectra. IR spectra were obtained employing a Shimadzu FTIR-8400 instrument with a Golden Gate™ attachment that uses a type IIa diamond as a single reflection element so that the IR spectrum of the compound (solid or liquid) could be detected directly (thin layer). High resolution mass spectra were recorded under FAB, ESI, EI and CI conditions by the analytical services at the University of Glasgow. Flash column chromatography was performed using forced flow of the indicated solvent system on EMD Geduran Silica Gel 60 as solid support and HPLC graded solvents as eluant. Reactions were monitored by thin layer chromatography (TLC) on Merck silica gel 60 covered aluminium sheets. TLC plates were developed under UV-light and/or with an acidic ethanolic anisaldehyde solution or a KMnO_4_-solution. All reagents were purchased from commercial suppliers and used without further purification unless otherwise stated.

### 2-Naphthyl boronic acid MIDA ester (7a)

Following a literature procedure,^13^ naphthyl boronic acid (0.86 g, 5.0 mmol) and MIDA (0.74 g, 5.0 mmol) were stirred in DMF (15 mL) at 85 °C for 16 h then allowed to cool to room temperature. The reaction mixture was diluted with EtOAc (100 mL) and H_2_O (100 mL) then separated. The organic phase was washed with H_2_O (50 mL) and brine (3 × 50 mL) then dried (Na_2_SO_4_), filtered and concentrated *in vacuo*. Purification by flash chromatography (diethyl ether : methanol, 98.5 : 1.5 then THF) gave a yellow oil. Addition of Et_2_O formed a white precipitate which was collected by vacuum filtration affording the title compound as a white solid (1.3 g, 93%). Analytical data were in accordance with literature values.^13^^1^H NMR (400 MHz, *d*_6_-DMSO) *δ* (ppm): 7.99 (1H, s, Ar-H), 7.94–7.88 (3H, m, Ar-H), 7.57–7.50 (3H, m, Ar-H), 4.38 (2H, d, *J* = 17.2 Hz, 2× NC*H*H), 4.17 (2H, d, *J* = 17.1 Hz, 2× NCH*H*), 2.52 (3H, s, NCH_3_).

### Neopentyl boronic acid MIDA ester (7l)

Neopentyl boronic acid (200 mg, 1.72 mmol) and MIDA (253 mg, 1.72 mmol) were stirred in DMF (5 mL) at 85 °C for 16 h then allowed to cool to room temperature. The reaction mixture was diluted with EtOAc (30 mL) and H_2_O (30 mL) then separated. The organic phase was washed 3 times with 5% LiCl solution then dried over Na_2_SO_4_, filtered and concentrated *in vacuo*. The residue was dissolved in a minimal amount of MeCN and dropped into ice-cold Et_2_O forming a white precipitate. The solvent was removed by filtration affording the title compound as a white solid (226 mg, 58%). ^1^H NMR (400 MHz, CDCl_3_) *δ* 3.76 (d, *J* = 16.2 Hz, 2H, NC*H*H), 3.60 (d, *J* = 16.1 Hz, 2H, NCH*H*), 2.84 (s, 3H, NCH_3_), 1.04 (s, 9H, 3× CH_3_), 0.58 (s, 2H, CH_2_); ^13^C NMR (100 MHz, CDCl_3_) *δ* 166.9 (2× C), 110.1 (CH_2_), 61.4 (2× CH_2_), 45.5 (CH_3_), 32.4 (3× CH_3_), 30.3 (C); IR (solid) 2949, 2862, 1739, 1464, 1109, 1032 cm^−1^; HRMS (ESI) exact mass calculated for C_10_H_18_NO_4_^10^BNa [M + Na]^+^*m*/*z* 249.1257, found *m*/*z* 249.1248.

### (*S*)-(4-(2-((*tert*-Butoxycarbonyl)amino)-3-methoxy-3-oxopropyl)phenyl)boronic acid MIDA ester (7m)

The boronic acid (200 mg, 0.620 mmol) and MIDA (91 mg, 0.62 mmol) were stirred in DMF (5 mL) at 85 °C for 16 h then allowed to cool to room temperature. The reaction mixture was diluted with EtOAc (30 mL) and H_2_O (30 mL) then separated. The organic phase was washed 3 times with 5% LiCl solution then dried over Na_2_SO_4_, filtered and concentrated *in vacuo*. The residue was dissolved in a minimal amount of MeCN and dropped into ice-cold Et_2_O forming a white precipitate. The solvent was removed by filtration affording the title compound as a white solid (223 mg, 82%).^1^H NMR (400 MHz, CDCl_3_) *δ* 7.45 (d, *J* = 7.8 Hz, 2H, Ar-H), 7.16 (d, *J* = 7.9 Hz, 2H, Ar-H), 5.00 (d, *J* = 8.1 Hz, 1H, NH), 4.62–4.52 (m, 1H, CH), 3.95 (d, *J* = 16.4 Hz, 2H, NC*H*H), 3.76 (dd, *J* = 16.4, 4.7 Hz, 2H, NCH*H*), 3.71 (s, 3H, OCH_3_), 3.13 (dd, *J* = 13.4, 5.4 Hz, 1H, C*H*H), 3.03 (dd, *J* = 13.4, 6.3 Hz, 1H, CH*H*), 2.54 (s, 3H, NCH_3_), 1.41 (s, 9H, 3× CH_3_); ^13^C NMR (100 MHz, CDCl_3_) *δ* 172.3 (C), 167.8 (2× C), 155.2 (C), 138.2 (C), 132.6 (2× CH), 129.5 (2× CH), 80.1 (C), 61.9 (2× CH_2_), 54.6 (CH), 52.4 (CH_3_), 47.7 (CH_3_), 38.5 (CH_2_), 28.4 (3× CH_3_). The ^13^C peak corresponding to the carbon atom adjacent to the boron atom was not observed. IR (thin film) 2980, 1743, 1705, 1612, 1500, 908 cm^−1^; HRMS (ESI) exact mass calculated for C_20_H_27_N_2_O_8_^10^BNa [M + Na]^+^*m*/*z* 457.1753, found *m*/*z* 457.1751.

### General procedure for allylation of MIDA boronates

A screw-top glass vial was charged with boronic acid MIDA ester (1 equiv.), dioxane (0.08 M), allyl bromide (2 equiv.) and Pd_2_dba_3_ (5 mol%). A 3 M aq. solution of K_3_PO_4_ (1 : 5 v/v ratio to dioxane) was added and the vial sealed under ambient atmosphere. The resulting mixture was heated to 60 °C by immersion of the entire vial into a preheated aluminium block until the substrate had been consumed, as judged by TLC analysis. The reaction mixture was cooled to room temperature, quenched with 1 M aq. NaOH then extracted with Et_2_O (3×). The combined organic extracts were dried (Na_2_SO_4_), filtered and concentrated *in vacuo*. Addition of 1,3,5-trimethoxybenzene (17 mg, 0.10 mmol) allowed determination of the yield by ^1^H NMR.

#### 2-Allylnaphthalene (9a)

The general procedure was employed for the allylation of 2-naphthylboronic acid MIDA ester (28 mg, 0.10 mmol). Yield determined by ^1^H NMR: 91%. Analytical data were in accordance with literature values.^14^^1^H NMR (400 MHz, CDCl_3_) *δ* 7.86–7.81 (3H, m, Ar-H), 7.67 (1H, s, Ar-H), 7.51–7.44 (2H, m, Ar-H), 7.37 (1H, dd, *J* = 8.4, 1.8 Hz, Ar-H), 6.10 (1H, ddt, *J* = 16.8, 10.1, 6.7 Hz, *CH*=CH_2_), 5.19 (1H, dq, *J* = 10.5, 1.7 Hz, C=*CHa*), 5.17–5.15 (1H, m, C=*CHb*), 3.59 (2H, d, *J* = 6.6 Hz, CH_2_).

#### 2-Allylfluorobenzene (9b)

The general procedure was employed for the allylation of 2-fluorophenylboronic acid MIDA ester (25 mg, 0.10 mmol). Yield determined by ^1^H NMR: 83%. Analytical data were in accordance with a commercial sample. ^1^H NMR (400 MHz, CDCl_3_) *δ* 7.22–7.16 (2H, m, Ar-H), 7.09–6.98 (2H, m, Ar-H), 6.01–5.91 (1H, m, C*H*=CH_2_), 5.10–5.05 (2H, m, CH=C*H*_2_), 3.41 (2H, dd, *J* = 6.6, 1.5 Hz, CH_2_).

#### 4-Allylchlorobenzene (9c)

The general procedure was employed for the allylation of 4-chlorophenylboronic acid MIDA ester (27 mg, 0.10 mmol). Yield determined by ^1^H NMR: 92%. Analytical data were in accordance with literature values.^15^^1^H NMR (400 MHz, CDCl_3_) *δ* 7.29–7.25 (2H, m, Ar-H), 7.14–7.11 (2H, m, Ar-H), 5.94 (1H, ddt, *J* = 17.2, 10.4, 6.8 Hz, *CH*=CH_2_), 5.11–5.10 (1H, m, CH=*CHa*), 5.09–5.05 (1H, m, CH=*CHb*), 3.36 (2H, d, *J* = 6.7 Hz, CH_2_).

#### 4-Allylbromobenzene (9d)

The general procedure was employed for the allylation of 4-bromophenylboronic acid MIDA ester (31 mg, 0.10 mmol). Yield determined by ^1^H NMR: 90%. Analytical data were in accordance with literature values.^15^^1^H NMR (400 MHz, CDCl_3_) *δ* 7.45–7.39 (2H, m, Ar-H), 7.09–7.04 (2H, m, Ar-H), 5.93 (1H, ddt, *J* = 16.6, 10.4, 6.4 Hz, *CH*=CH_2_), 5.11–5.03 (2H, m, CH=*CH*_2_), 3.34 (2H, d, *J* = 6.6 Hz, CH_2_).

#### 3-Allyliodobenzene (9e)

The general procedure was employed for the allylation of 3-iodophenylboronic acid MIDA ester (36 mg, 0.10 mmol). Yield determined by ^1^H NMR: 91%. Analytical data were in accordance with literature values.^16^^1^H NMR (400 MHz, CDCl_3_) *δ* 7.57–7.53 (2H, m, Ar-H), 7.17–7.14 (1H, m, Ar-H), 7.06–7.00 (1H, m, Ar-H), 5.92 (1H, ddt, *J* = 18.0, 10.7, 6.7 Hz, *CH*=CH_2_), 5.13–5.06 (2H, m, CH=*CH*_2_), 3.33 (2H, d, *J* = 6.6 Hz, CH_2_).

#### 3-Allylnitrobenzene (9f)

The general procedure was employed for the allylation of 3-nitrophenylboronic acid MIDA ester (83 mg, 0.30 mmol). Yield determined by ^1^H NMR: 92%. Analytical data were in accordance with literature values.^17^^1^H NMR (400 MHz, CDCl_3_) *δ* 8.08–8.06 (2H, m, Ar-H), 7.54–7.51 (1H, m, Ar-H), 7.46 (1H, ddd, *J* = 7.6, 7.0, 1.6 Hz, Ar-H), 5.96 (1H, ddt, *J* = 16.8, 10.1, 6.7 Hz, *CH*=CH_2_), 5.17 (1H, dq, *J* = 10.3, 1.4 Hz, CH=*CHa*), 5.13 (1H, dq, *J* = 17.3, 1.6 Hz, CH=*CHb*), 3.50 (2H, d, *J* = 6.6 Hz, CH_2_).

#### 2-Allylcyanobenzene (9g)

The general procedure was employed for the allylation of 2-cyanophenylboronic acid MIDA ester (77 mg, 0.30 mmol). Yield determined by ^1^H NMR: 88%. Analytical data were in accordance with literature values.^18^^1^H NMR (400 MHz, CDCl_3_) *δ* 7.63 (1H, dd, *J* = 7.7, 1.1 Hz, Ar-H), 7.53 (1H, td, *J* = 3.9, 1.4 Hz, Ar-H), 7.36–7.28 (2H, m, Ar-H), 5.95 (1H, ddt, *J* = 16.9, 10.2, 6.6 Hz, *CH*=CH_2_), 5.17–5.08 (2H, m, CH=*CH*_2_), 3.61 (2H, d, *J* = 6.6 Hz, CH_2_).

#### 2-Phenyl-1,4-pentadiene (9h)

The general procedure was employed for the allylation of 1-phenylvinylboronic acid MIDA ester (78 mg, 0.30 mmol). Yield determined by ^1^H NMR: 89%. Analytical data were in accordance with literature values.^19^^1^H NMR (400 MHz, CDCl_3_) *δ* 7.47–7.42 (2H, m, Ar-H), 7.36–7.30 (2H, m, Ar-H), 7.30–7.25 (1H, m, Ar-H), 5.91 (1H, ddt, *J* = 17.1, 10.1, 6.6 Hz, *CH*=CH_2_), 5.42–5.41 (1H, m, C=*CHa*), 5.16–5.04 (3H, m, C=*CHb*, CH=*CH*_2_), 3.27–3.25 (2H, m, CH_2_).

#### 4-Allylphenol (9i)

The general procedure was employed for the allylation of 4-hydroxyphenylboronic acid MIDA ester (31 mg, 0.10 mmol), except that K_3_PO_4_ (aq.) was replaced with 3 M aq. NaHCO_3_. Yield determined by ^1^H NMR: 28%. Analytical data were in accordance with literature values.^20^^1^H NMR (400 MHz, CDCl_3_) *δ* 7.06 (2H, d, *J* = 8.4 Hz, Ar-H), 6.78 (2H, d, *J* = 8.5 Hz, Ar-H), 5.95 (1H, ddt, *J* = 16.7, 10.4, 6.7 Hz, *CH*=CH_2_), 5.08–5.03 (2H, m, CH=*CH*_2_), 4.86 (1H, s, OH), 3.32 (2H, d, *J* = 6.7 Hz, CH_2_).

#### 2-Allylfuran (9j)

The general procedure was employed for the allylation of 2-furanylboronic acid MIDA ester (22 mg, 0.10 mmol), except that the reaction was run at room temperature. Yield determined by ^1^H NMR: 45%. Analytical data were in accordance with literature values.^21^^1^H NMR (400 MHz, CDCl_3_) *δ* 7.41 (1H, d, *J* = 2.4 Hz, Ar-H), 7.32 (1H, d, *J* = 2.8 Hz, Ar-H), 6.29 (1H, d, *J* = 2.8 Hz, Ar-H), 6.02 (1H, d, *J* = 3.1 Hz, Ar-H), 5.97–5.88 (1H, m, *CH*=CH_2_), 5.16–5.10 (2H, m, CH=*CH*_2_), 3.39 (2H, d, *J* = 6.5 Hz, CH_2_).

#### 2-Allyl-*N*-Boc pyrrole (9k)

The general procedure was employed for the allylation of *N*-Boc-pyrrole-2-boronic acid MIDA ester (32 mg, 0.10 mmol). Yield determined by ^1^H NMR: 67%. Analytical data were in accordance with literature values.^22^^1^H NMR (400 MHz, CDCl_3_) *δ* 7.22 (1H, dd, *J* = 1.8, 1.5 Hz, Ar-H), 6.09 (1H, t, *J* = 3.3 Hz, Ar-H), 6.02 (1H, ddt, *J* = 16.7, 10.2, 6.4 Hz, *CH*=CH_2_), 5.98–5.96 (1H, m, Ar-H), 5.10–5.02 (2H, m, CH=*CH*_2_), 3.61 (2H, dd, *J* = 6.3, 1.2 Hz, CH_2_), 1.58 (9H, s, C(CH_3_)_3_).

#### (*E*)-1-Phenyl-5,5-dimethylhexene (9l)

A 4 mL screw-top glass vial was charged with neopentyl boronic acid MIDA ester (45 mg, 0.20 mmol), benzene (1.25 mL), cinnamyl chloride (0.030 mL, 0.30 mmol) and Pd_2_dba_3_ (10 mg, 0.011 mmol) and was stirred vigorously. A 3 M aq. solution of K_3_PO_4_ (0.5 mL) was added and the vial was sealed under ambient atmosphere. The resulting mixture was heated to 60 °C by immersion of the entire vial into a preheated aluminium block until the substrate had been consumed, as judged by TLC analysis. The reaction mixture was cooled to room temperature, quenched with 1 M aq. NaOH (10 mL) then extracted with Et_2_O (3 × 10 mL). The combined organic extracts were dried (Na_2_SO_4_), filtered and concentrated carefully *in vacuo*. Addition of 1,3,5-trimethoxybenzene (17 mg, 0.10 mmol) allowed determination of the yield by ^1^H NMR: 58% ^1^H NMR (400 MHz, CDCl_3_) *δ* 7.37–7.27 (m, 4H, Ar-H), 7.23–7.17 (m, 1H, Ar-H), 6.40 (d, *J* = 15.8 Hz, 1H, C*H*-Ph), 6.25 (dt, *J* = 15.8, 6.6 Hz, 1H, C*H*–CH_2_), 2.25–2.16 (m, 2H, CH_2_), 1.42–1.34 (m, 2H, CH_2_), 0.95 (s, 9H, 3× CH_3_); ^13^C NMR (100 MHz, CDCl_3_) *δ* 138.1 (C), 132.0 (CH), 129.4 (CH), 128.6 (2× CH), 126.8 (2× CH), 126.0 (CH), 43.8 (CH_2_), 30.6 (C), 29.5 (3× CH_3_), 28.6 (CH_2_); HRMS (EI) exact mass calculated for C_14_H_20_ [M]^+^*m*/*z* 188.1565, found *m*/*z* 188.1557.

#### l-Phenylalanine-*N*-[(1,1-dimethylethoxy)carbonyl]-4-[(2*E*)-3-phenyl-2-propen-1-yl]-, methyl ester (9m)

The general procedure was employed for the allylation of boronic MIDA ester (43 mg, 0.10 mmol) using cinnamyl chloride (0.030 mL, 0.20 mmol) except for that dioxane was changed for 1.25 mL of benzene. Purification by silica chromatography using 4% ethyl acetate in petroleum ether afforded the title compound as a colourless oil (39 mg, 99%). Analytical data were in accordance with literature values.^23^^1^H NMR (400 MHz, CDCl_3_) *δ* 7.38–7.33 (m, 2H, Ar-H), 7.32–7.27 (m, 2H, Ar-H), 7.23–7.15 (m, 3H, Ar-H), 7.09–7.03 (m, 2H, Ar-H), 6.45 (d, *J* = 15.8 Hz, 1H, CH_2_CH=C*H*), 6.33 (dt, *J* = 15.8, 6.7 Hz, 1H, CH_2_C*H*=CH), 4.96 (d, *J* = 8.4 Hz, 1H, NH), 4.58 (q, *J* = 6.8 Hz, 1H, CH), 3.72 (s, 3H, OCH_3_), 3.52 (d, *J* = 6.8 Hz, 2H, CH_2_), 3.06 (m, 2H, CH_2_), 1.41 (s, 9H, 3× CCH_3_). ^13^C NMR (101 MHz, CDCl_3_) *δ* 172.5 (C), 155.2 (C), 139.1 (C), 137.6 (C), 133.9 (C), 131.3 (CH), 129.6 (2× CH), 129.2 (CH), 129.0 (2× CH), 128.6 (2× CH), 127.3 (CH), 126.3 (2× CH), 80.1 (C), 54.6 (CH), 52.3 (CH_3_), 39.1 (CH_2_), 38.1 (CH_2_), 28.5 (3× CH_3_). IR (thin film): 1719, 1366, 1165, 692 cm^−1^. HRMS (ESI) exact mass calculated for C_24_H_29_NO_4_Na [M + Na]^+^*m*/*z* 418.1989, found *m*/*z* 418.1979.

#### 2-(2-Methyl-2-propen-1-yl)-naphthalene (11a)

The general procedure was employed for the allylation of 2-naphthylboronic acid MIDA ester (85 mg, 0.30 mmol) using 2-methylallyl bromide (0.030 mL, 0.30 mmol). Purification by flash chromatography (petroleum ether) afforded the title compound as a colourless oil (47.2 mg, 86%). Analytical data were in accordance with literature values.^24^^1^H NMR (500 MHz, CDCl_3_) *δ* (ppm): 7.83–7.78 (3H, m, Ar-H), 7.65 (1H, s, Ar-H), 7.45 (2H, quintet d, *J* = 6.8, 1.5 Hz, Ar-H), 7.35 (1H, dd, *J* = 8.4, 1.7 Hz, Ar-H), 4.88–4.87 (1H, m, C=*CHa*), 4.81–4.80 (1H, m, C=*CHb*), 3.50 (2H, s, CH_2_), 1.72 (3H, s, CH_3_).

#### 2-(3-Phenyl-2-propen-1-yl)-naphthalene (11b)

The general procedure was employed for the allylation of 2-naphthylboronic acid MIDA ester (32 mg, 0.10 mmol) using cinnamyl chloride (0.030 mL, 0.20 mmol). Purification by flash chromatography (petroleum ether) afforded the title compound as a colourless oil (20.2 mg, 83%). Analytical data were in accordance with literature values.^25^^1^H NMR (400 MHz, CDCl_3_) *δ* 7.85–7.80 (3H, m, Ar-H), 7.70 (1H, s, Ar-H), 7.50–7.43 (2H, m, Ar-H), 7.41–7.38 (3H, m, Ar-H), 7.34–7.29 (2H, m, Ar-H), 7.25–7.21 (1H, m, Ar-H), 6.53 (1H, d, *J* = 15.8 Hz, CH_2_CH=*CH*), 6.45 (1H, dt, *J* = 15.8, 6.3 Hz, CH_2_*CH*=CH), 3.73 (2H, d, *J* = 6.2 Hz, *CH*_2_CH=CH).

#### 2-(2-Buten-1-yl)-naphthalene/2-(1-methyl-2-propen-1-yl)-naphthalene ((major) 11c/11c* (minor))

The general procedure was employed for the allylation of 2-naphthylboronic acid MIDA ester (32 mg, 0.10 mmol) using 3-chlorobut-1-ene (0.020 mL, 0.20 mmol). Purification by flash chromatography (petroleum ether) afforded the title compounds as a colourless oil (12.4 mg, 68%, 1.6 : 1). Analytical data were in accordance with literature values.^24^^1^H NMR (400 MHz, CDCl_3_) *δ* 7.82–7.77 (3H, m, Ar-H), 7.66 (1H, s, Ar-H), 7.62 (1H, s, Ar-H), 7.48–7.40 (2H, m, Ar-H), 7.39–7.32 (1H, m, Ar-H), 6.10 (1H, ddd, *J* = 16.9, 10.3, 6.4 Hz, *CH*=CH_2_), 5.72–5.54 (2H, m, *CH*=*CH*), 5.14–5.08 (2H, m, CH=*CH*_2_), 3.67–3.57 (1H, m, *CH*CH_3_), 3.49 (2H, dd, *J* = 6.5, 1.2 Hz, CH_2_), 1.73 (3H, dq, *J* = 6.1, 1.4 Hz, CH*CH*_3_), 1.47 (3H, d, *J* = 7.0 Hz, CH=CH*CH*_3_).

#### 2-(3-Methyl-2-buten-1-yl)-naphthalene/2-(2-methyl-3-buten-2-yl)-naphthalene ((major) 11d/11d* (minor))

The general procedure was employed for the allylation of 2-naphthylboronic acid MIDA ester (32 mg, 0.10 mmol) using prenyl chloride (0.020 mL, 0.20 mmol). Purification by flash chromatography (petroleum ether) afforded the title compounds as a colourless oil (15.1 mg, 77%, 3 : 1). Analytical data were in accordance with literature values.^26^^1^H NMR (400 MHz, CDCl_3_) *δ* 0.82 (3H, m, Ar-H), 7.62 (1H, s, Ar-H), 7.52–7.40 (2H, m, Ar-H), 7.34 (1H, dd, *J* = 8.5, 1.8 Hz, Ar-H), 6.12 (1H, dd, *J* = 17.3, 10.7 Hz, *CH*=CH_2_), 5.43 (1H, septet, *J* = 7.3, 1.4 Hz, *CH*=C(CH_3_)_2_), 5.15–5.10 (2H, m, CH=*CH*_2_), 3.52 (2H, d, *J* = 7.3 Hz, CH_2_), 1.79 (6H, dd, *J* = 4.4, 1.4 Hz, CH=*C*(*CH*_3_)_2_), 1.52 (6H, s, 2× CH_3_).

#### 2-(2-Propen-1-yl-3,3-d_2_)-naphthalene/2-(2-propen-1-yl-1,1-d_2_)-naphthalene (13/14)

The general procedure was employed for the allylation of 2-naphthylboronic acid MIDA ester (28 mg, 0.10 mmol) except that Pd_2_dba_3_ was replaced by Pd(OAc)_2_ (5 mol%). Coupling with freshly prepared dideuteroallyl bromide^27^ (0.23 mL, 0.89 M in Et_2_O, 0.20 mmol) was followed by purification by flash chromatography (petroleum ether) to afford the title compounds as a colourless oil (14.1 mg, 83%, 1 : 1 isomer ratio). Analytical data were in accordance with literature values.^28^^1^H NMR (400 MHz, CDCl_3_) *δ* 7.83–7.78 (3H, m, Ar-H), 7.64 (1H, dd, *J* = 1.8, 0.8 Hz, Ar-H), 7.49–7.42 (2H, m, Ar-H), 7.35 (1H, dd, *J* = 8.4, 1.8 Hz, Ar-H), 6.09–6.02 (1H, m, C*H*=CH_2_/C*H*=CD_2_), 5.16 (0.5H, dd, *J* = 12.0, 2.0 Hz, CH=C*H*H), 5.13 (0.5H, dd, *J* = 5.2, 2.0 Hz, CH=CH*H*), 3.57 (1H, dd, *J* = 6.6, 0.7 Hz, C*H*_2_CH=CD_2_); ^13^C NMR (100 MHz, CDCl_3_) *δ* 137.7 (2× C), 137.4 (CH), 137.2 (CH), 133.8 (2× C), 132.3 (2× C), 128.0 (2× CH), 127.8 (2× CH), 127.6 (2× CH), 127.5 (2× CH), 126.8 (2× CH), 126.1 (2× CH), 125.4 (2× CH), 116.2 (CH_2_), 115.7 (p, ^1^*J*(C-D) = 23.7 Hz, CD_2_), 40.4 (CH_2_), 39.8 (p, ^1^*J*(C-D) = 19.6 Hz, CD_2_); IR (thin film) 3053, 2923, 2113, 1689 cm^−1^; HRMS (EI) exact mass calculated for C_13_H_10_D_2_ [M]^+^*m*/*z* 170.1059, found *m*/*z* 170.1080.

#### *tert*-Butyl 2-(4-phenyl boronic acid MIDA ester)-propionate (16)

Following a literature procedure,^11^ to a dry 2-necked flask with a condenser was added zinc powder (1.2 g, 19 mmol), THF (7.5 mL) and trimethylsilyl chloride (0.080 mL, 0.63 mmol). The mixture was stirred for 15 minutes. A solution of *tert*-butyl 2-bromopropionate (2.10 mL, 12.5 mmol) in THF (17.5 mL) was added over 2–3 minutes with an exotherm observed. Once the mixture returned to room temperature, stirring was discontinued. The solution was used directly in the next step (assumed concentration: 0.23 M). To a dry flask charged with a stir bar, was added 4-bromophenylboronic acid MIDA ester (0.31 g, 1.0 mmol), Pd(dba)_2_ (5.8 mg, 0.010 mmol), QPhos (7.1 mg, 0.010 mmol) and THF (3.0 mL). The mixture was stirred briefly, then a freshly prepared solution of *tert*-butyl 2-bromozincpropionate in THF (8.7 mL, 2.0 mmol) was added. The reaction mixture was stirred at 65 °C for 1.5 h. After cooling to room temperature, the reaction was quenched with sat. aq. NH_4_Cl (10 mL), followed by addition of brine (10 mL). The phases were separated, and the organic phase was dried (Na_2_SO_4_), filtered and concentrated *in vacuo*. The residue was adsorbed onto silica gel then purification by flash chromatography (diethyl ether : methanol, 98.5 : 1.5 then THF) afforded the title compound as a light pink solid (0.32 g, 90%).^1^H NMR (400 MHz, *d*_6_-DMSO) *δ* 7.39 (2H, d, *J* = 8.1 Hz, Ar-H), 7.25 (2H, d, *J* = 8.1 Hz, Ar-H), 4.32 (2H, d, *J* = 17.2 Hz, 2× NC*H*H), 4.11 (2H, d, *J* = 17.2 Hz, 2× NCH*H*), 3.64 (1H, q, *J* = 7.1 Hz, C*H*CH_3_), 2.49 (3H, s, NCH_3_), 1.40 (3H, s, CH_3_), 1.34 (9H, s, 3× CH_3_); ^13^C NMR (100 MHz, *d*_6_-DMSO) *δ* 174.3 (C), 173.0 (C), 169.3 (2× C), 141.6 (C), 132.5 (2× CH), 126.5 (2× CH), 79.9 (C), 61.8 (CH_2_), 47.5 (CH_3_), 45.5 (CH), 27.6 (3× CH_3_), 18.5 (CH_3_); IR (solid) 2978, 1724, 1713, 1367, 1150 cm^−1^; HRMS (ESI) exact mass calculated for C_18_H_24_BNNaO_6_ [M + Na]^+^*m*/*z* 384.1589, found *m*/*z* 384.1575.

#### *tert*-Butyl 2-(4-methallylphenyl)-propionate (17)

The general procedure was employed for the allylation of *tert*-butyl MIDA (36 mg, 0.10 mmol) using 2-methylallyl bromide (0.020 mL, 0.20 mmol). Yield was determined by ^1^H NMR: 73%. ^1^H NMR (400 MHz, CDCl_3_) *δ* 7.21 (2H, d, *J* = 8.1 Hz, Ar-H), 7.13 (2H, d, *J* = 8.1 Hz, Ar-H), 4.81–4.79 (1H, m, C=C*H*H), 4.73–4.71 (1H, m, C=CH*H*), 3.59 (1H, q, *J* = 7.2 Hz, C*H*CH_3_), 3.29 (2H, s, Ar-CH_2_C), 1.67 (3H, s, CCH_3_), 1.44 (3H, d, *J* = 7.2 Hz, CH*CH*_3_), 1.39 (9H, s, 3× CCH_3_); ^13^C NMR (100 MHz, CDCl_3_) *δ* 173.9 (C), 145.2 (C), 139.0 (C), 138.3 (C), 128.9 (2× CH), 127.3 (2× CH), 111.6 (CH_2_), 80.3 (C), 46.0 (CH), 44.1 (CH_2_), 27.8 (3× CH_3_), 21.9 (CH_3_), 18.3 (CH_3_); IR (thin film) 2978, 2934, 1728, 1452, 1148, 739 cm^−1^; HRMS (ESI) exact mass calculated for C_17_H_24_NaO_2_ [M + Na]^+^*m*/*z* 283.1669, found *m*/*z* 283.1666.

#### Ibuprofen (18)

A stirred solution of ester (18 mg, 0.069 mmol), palladium acetate (1.1 mg, 0.0050 mmol) and DavePhos (3.9 mg, 0.010 mmol) in dioxane/H_2_O (1.25 mL/0.25 mL) under an argon atmosphere was evacuated and backfilled with H_2_. The reaction mixture was stirred at room temperature under a H_2_ atmosphere for 16 h. The flask was evacuated and backfilled with argon, then conc. HCl (0.60 mL) was added to give an overall concentration of 4 M HCl. The reaction mixture was stirred at room temperature for 16 h then quenched with 2 M aq. NaOH. The mixture was extracted with Et_2_O (2 × 10 mL) then the aqueous phase adjusted to pH 1 with 2 M aq. HCl. The aqueous phase was extracted with Et_2_O (2 × 10 mL) then the combined organic extracts were dried (Na_2_SO_4_), filtered and concentrated *in vacuo* affording the title compound as a white solid (9.5 mg, 67%). Analytical data were in accordance with literature values.^29^^1^H NMR (400 MHz, CDCl_3_) *δ* 8.62 (1H, s, COOH), 7.22 (2H, d, *J* = 7.9 Hz, Ar-H), 7.10 (2H, d, *J* = 7.8 Hz, Ar-H), 3.71 (1H, q, *J* = 7.1 Hz, *CH*CH_3_), 2.44 (2H, d, *J* = 7.2 Hz, CH_2_), 1.84 (1H, septet, *J* = 6.6 Hz, *CH*(CH_3_)_2_), 1.50 (3H, d, *J* = 7.1 Hz, CH*CH*_3_), 0.90 (6H, d, *J* = 6.6 Hz, CH(*CH*_3_)_2_).

## Conflicts of interest

There are no conflicts to declare.

## Supplementary Material

RA-010-D0RA03338C-s001
