# Supplementary material for: MIDA boronate allylation – synthesis of ibuprofen
Source: RSC Adv. 2020 Aug 18;10(51):30624–30. doi: 10.1039/d0ra03338c (PMC9056333; doi:10.1039/d0ra03338c)
Supplement: RA-010-D0RA03338C-s001 [file RA-010-D0RA03338C-s001.pdf]

Electronic Supplementary Material (ESI) for RSC Advances.
This journal is © The Royal Society of Chemistry 2020

Supplementary Appendix – NMR Spectra

MIDA Boronate Allylation – Synthesis of Ibuprofen

David Phillips, Glen Brodie, Sarah Memarzadeh, Gi Lum Tang, David J. France

School of Chemistry, University of Glasgow, Glasgow, G12 8QQ, UK.

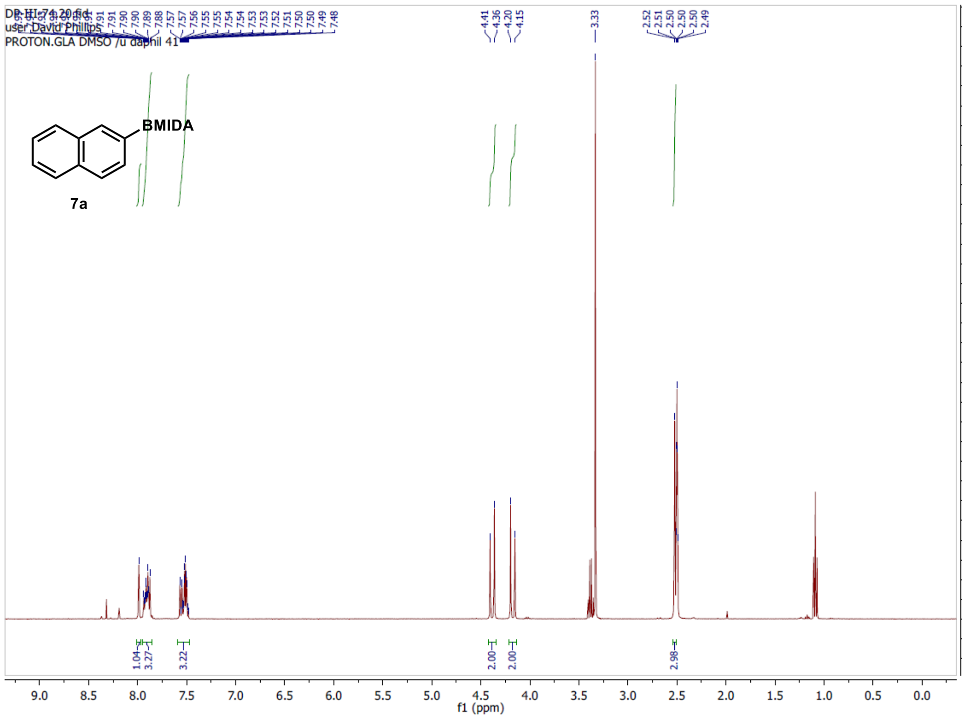

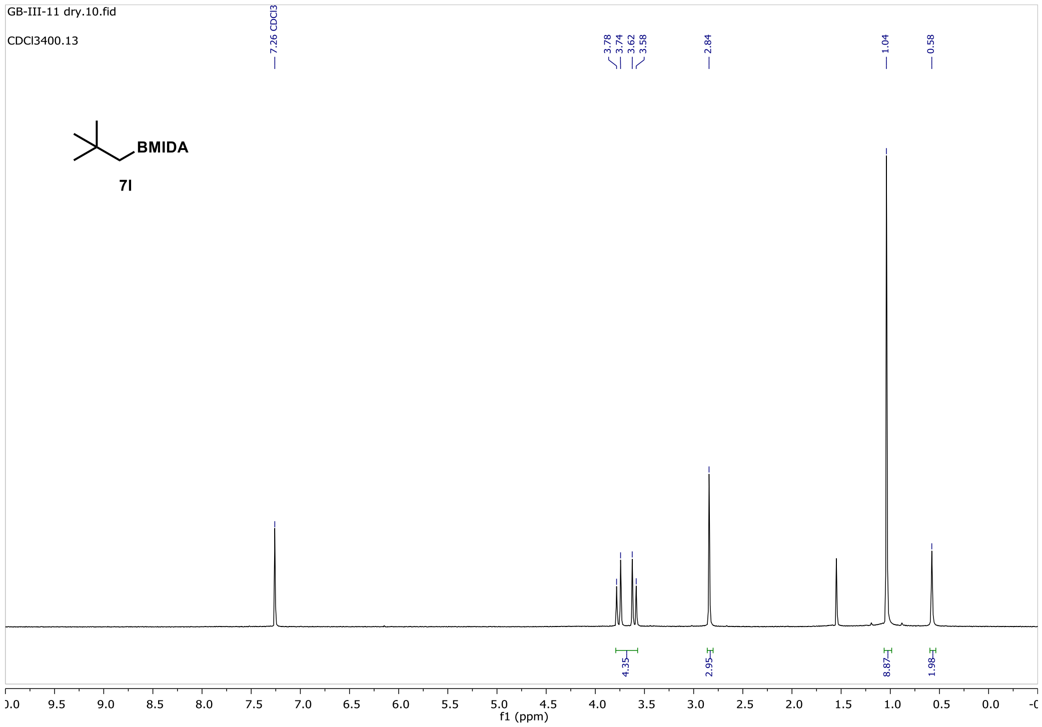
GB-III-11 dry.10.fid
CDCl3400.13
7.26 CDCl3
3.78
3.74
3.62
3.58
2.84
1.04
0.58
BMIDA
7l
4.35
2.95
8.87
1.98
f1 (ppm)

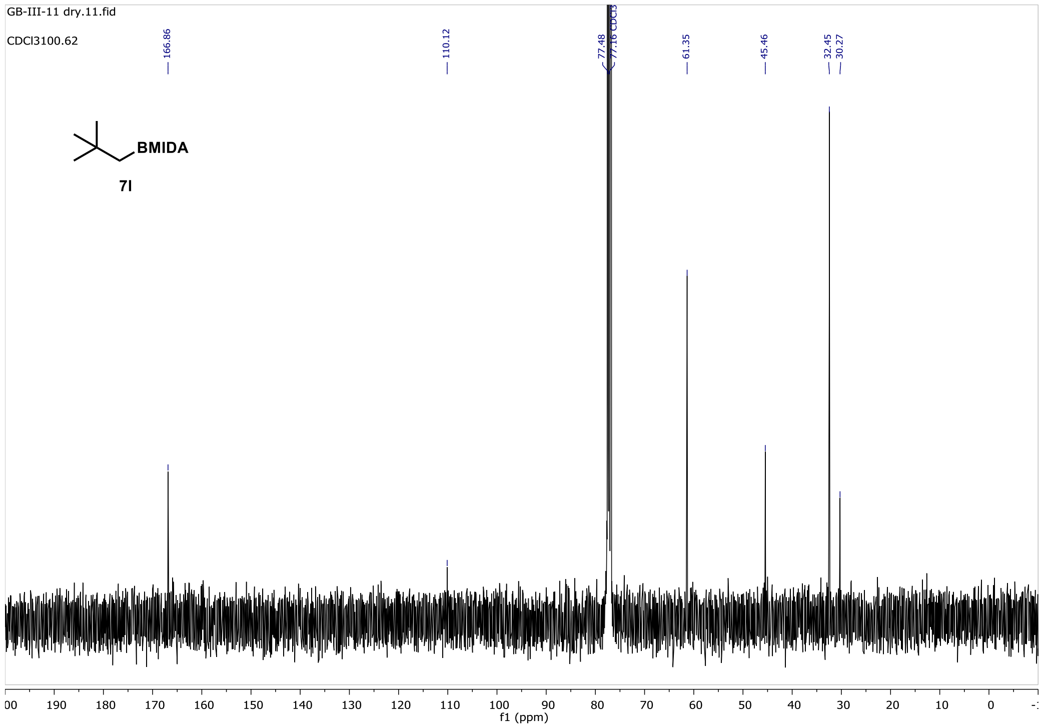
GB-III-11 dry.11.fid
CDCl3100.62
166.86
110.12
77.48
77.16 CDCl3
61.35
45.46
32.45
30.27
BMIDA
7l
f1 (ppm)

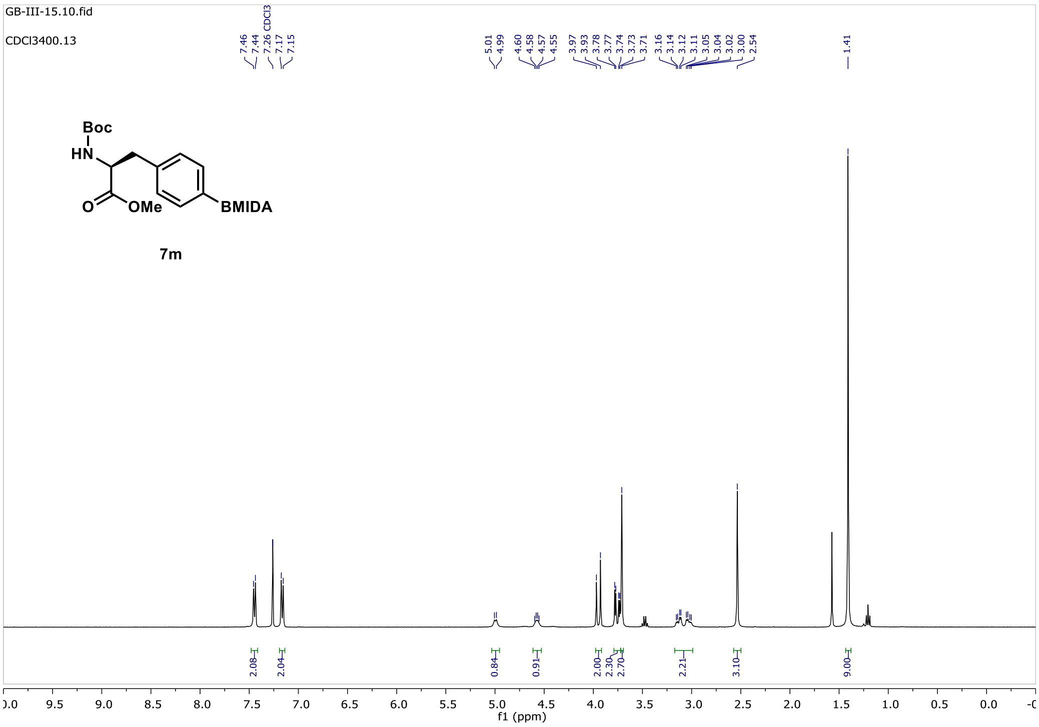
GB-III-15.10.fid
CDCl3400.13
7.46
7.44
7.26 CDCl3
7.17
7.15
5.01
4.99
4.60
4.58
4.57
4.55
3.97
3.93
3.78
3.77
3.74
3.73
3.71
3.16
3.14
3.12
3.11
3.05
3.04
3.02
3.00
2.54
1.41
Boc
HN
O
OMe
BMIDA
7m
2.08
2.04
0.84
0.91
2.00
2.30
2.70
2.21
3.10
9.00
f1 (ppm)

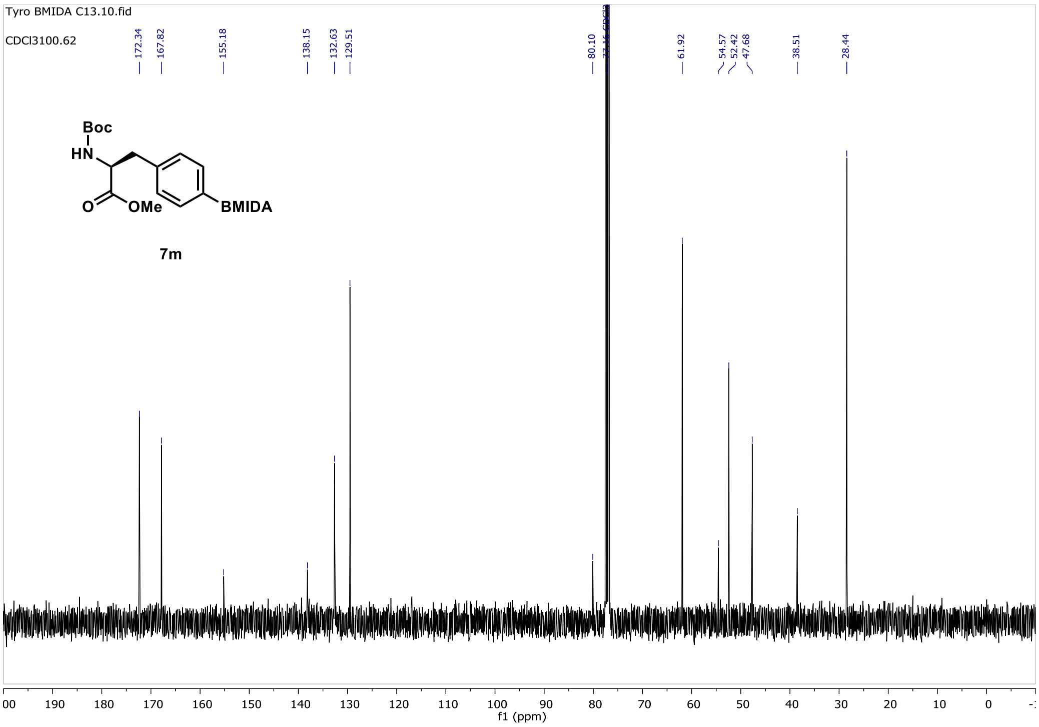
Tyro BMIDA C13.10.fid
CDCl3100.62
172.34
167.82
155.18
138.15
132.63
129.51
80.10
61.92
54.57
52.42
47.68
38.51
28.44
Boc
HN
O
OMe
BMIDA
7m
f1 (ppm)

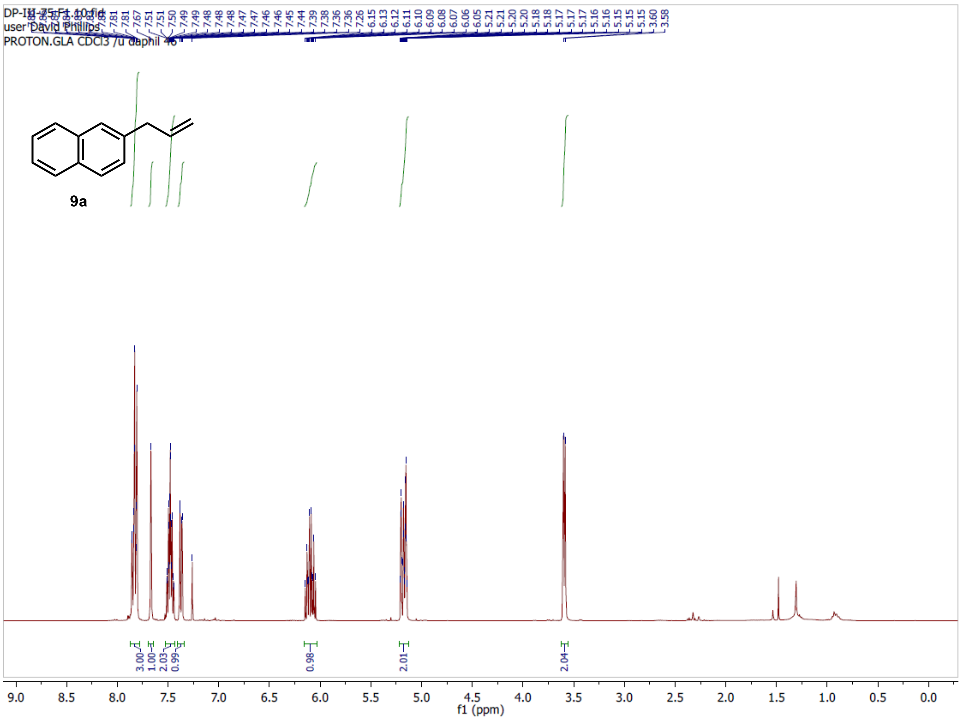
DP-III-75 F1.10.fid
user David Phillips
PROTON.GLA CDCl3 /u daphil 46
9a
f1 (ppm)

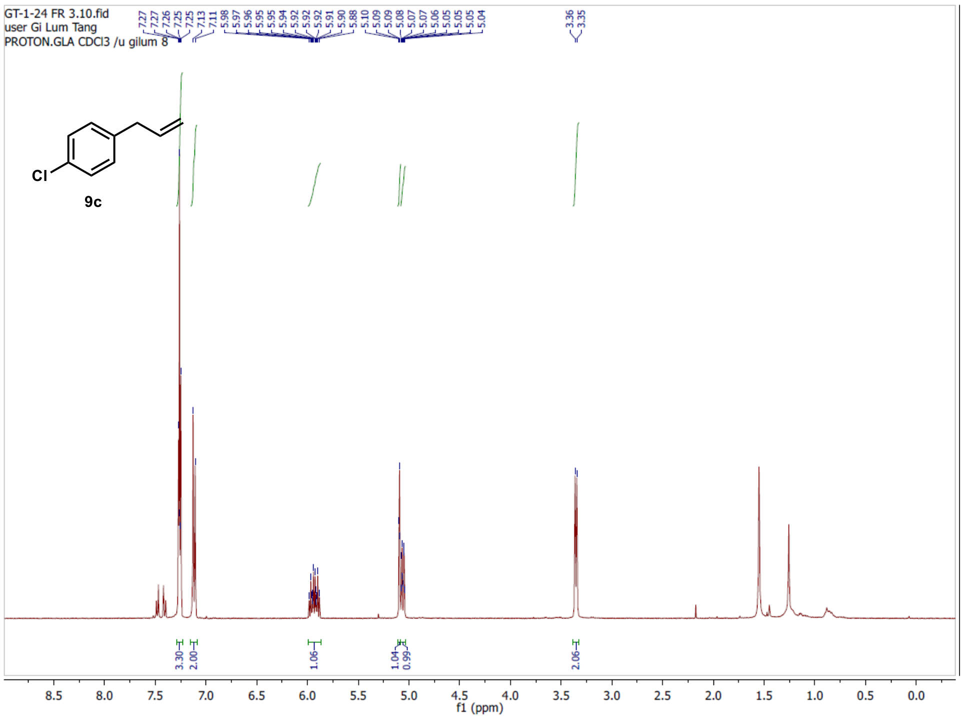
GT-1-24 FR 3.10.fid
user Gi Lum Tang
PROTON.GLA CDCl3 /u gilum 8
Cl
9c
f1 (ppm)

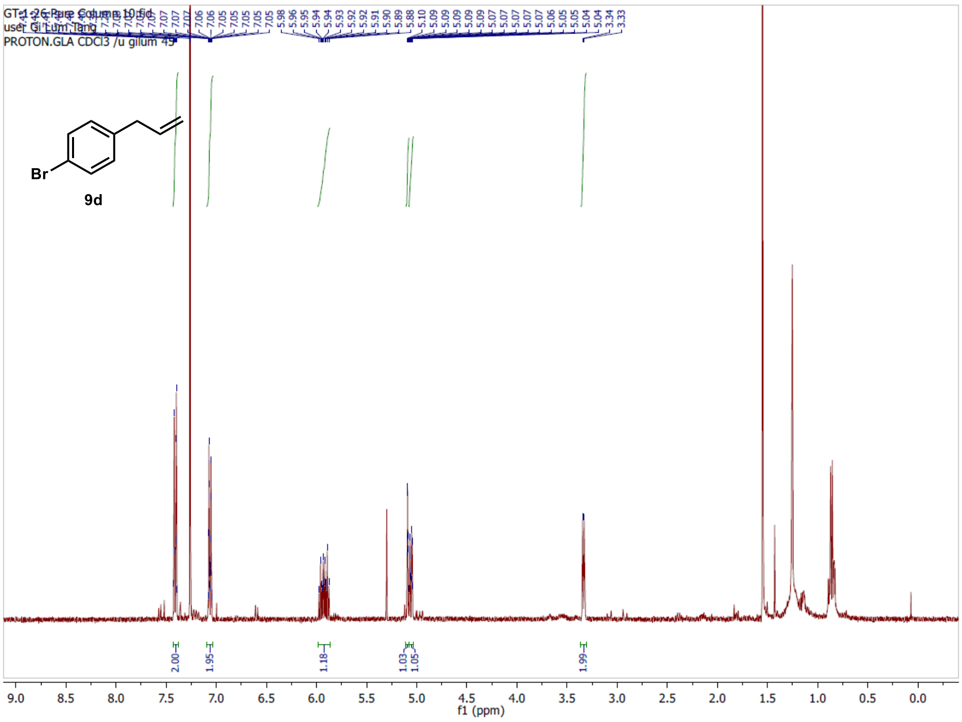
user Gi Lum Tang
PROTON.GLA CDCl3 /u gilum 45
Br
9d
2.00
1.95
1.18
1.03
1.05
1.99
9.0 8.5 8.0 7.5 7.0 6.5 6.0 5.5 5.0 4.5 4.0 3.5 3.0 2.5 2.0 1.5 1.0 0.5 0.0
f1 (ppm)

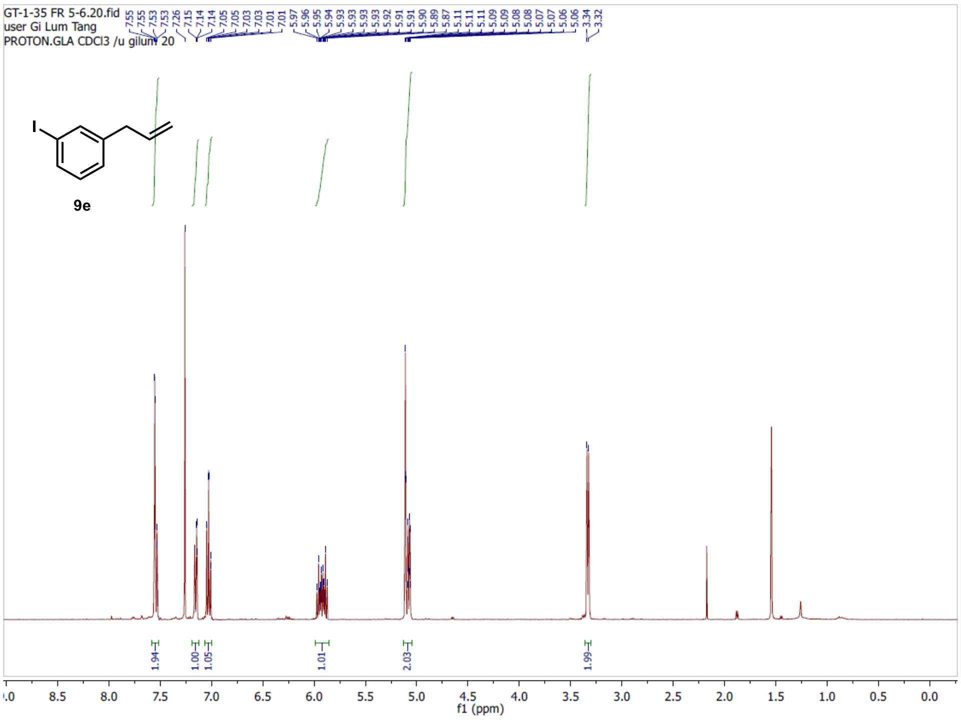
GT-1-35 FR 5-6.20.fid
user Gi Lum Tang
PROTON.GLA CDCl3 /u gilum 20
I
9e
1.94
1.00
1.05
1.01
2.03
1.99
8.5 8.0 7.5 7.0 6.5 6.0 5.5 5.0 4.5 4.0 3.5 3.0 2.5 2.0 1.5 1.0 0.5 0.0
f1 (ppm)

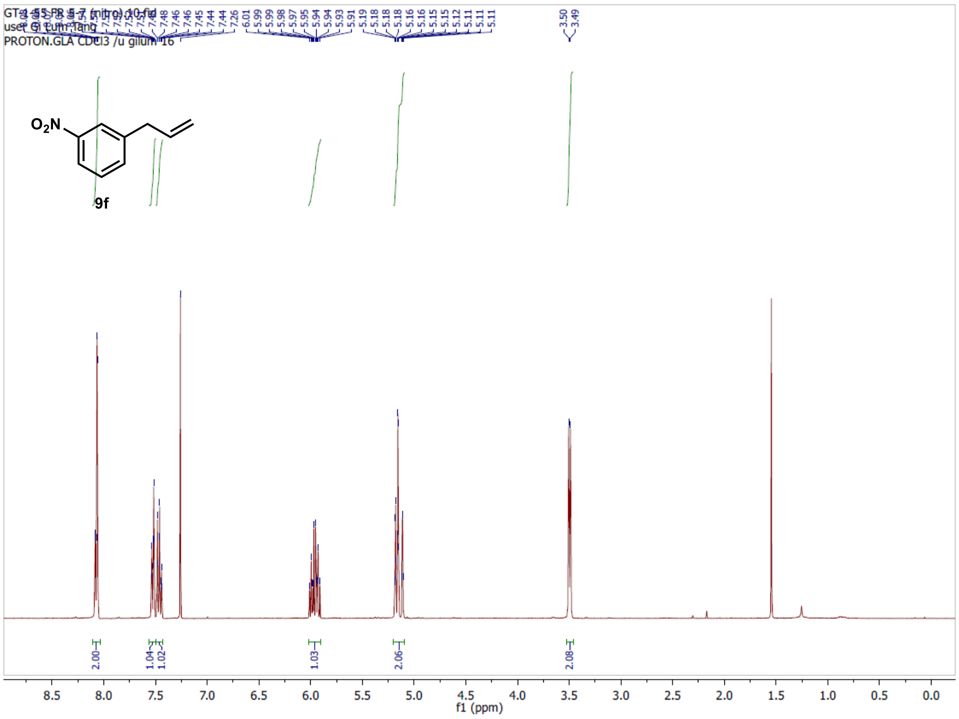
PROTON.GLA CDCl3 /u gilum 16
O2N
9f
2.00
1.04
1.02
1.03
2.06
2.08
8.5 8.0 7.5 7.0 6.5 6.0 5.5 5.0 4.5 4.0 3.5 3.0 2.5 2.0 1.5 1.0 0.5 0.0
f1 (ppm)

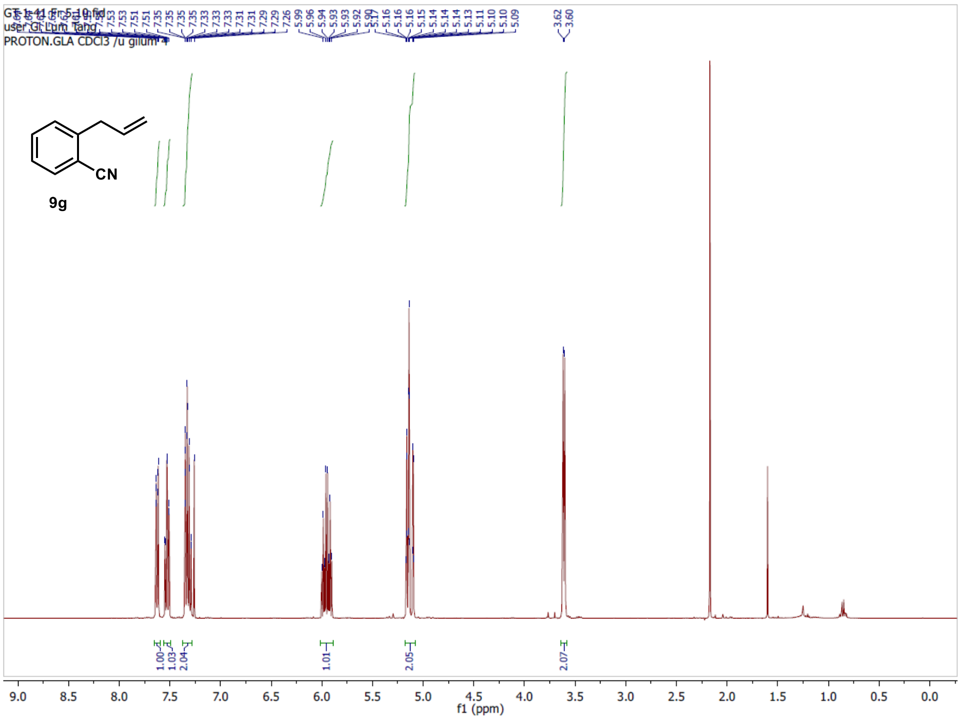
PROTON.GLA CDCl3 /u gilum 4
CN
9g
1.00
1.03
2.04
1.01
2.05
2.07
9.0 8.5 8.0 7.5 7.0 6.5 6.0 5.5 5.0 4.5 4.0 3.5 3.0 2.5 2.0 1.5 1.0 0.5 0.0
f1 (ppm)

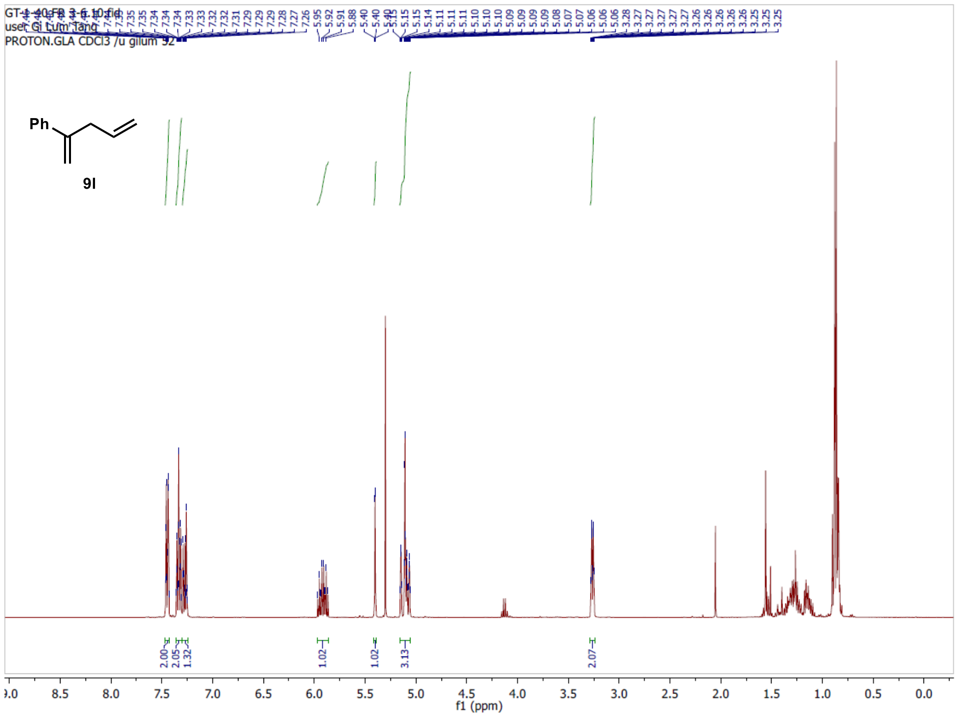
GT-I-40.FB 3-6.10.fid
user Gi Lum Tang
PROTON.GLA CDCl3 /u gilum 92
Ph
9l
f1 (ppm)

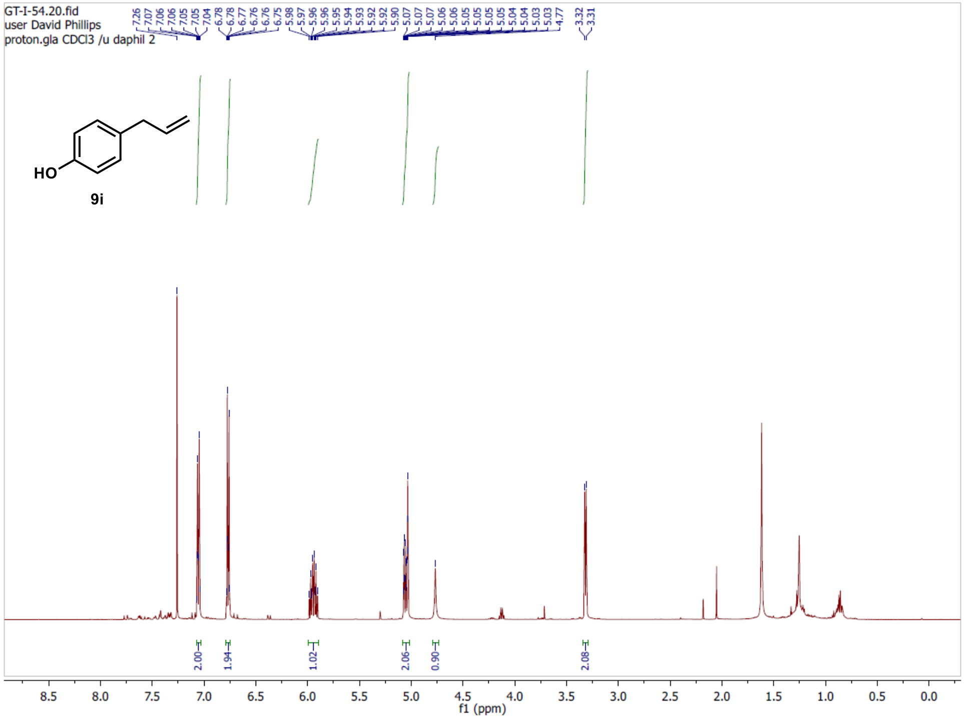
GT-I-54.20.fid
user David Phillips
proton.gla CDCl3 /u daphil 2
HO
9i
f1 (ppm)

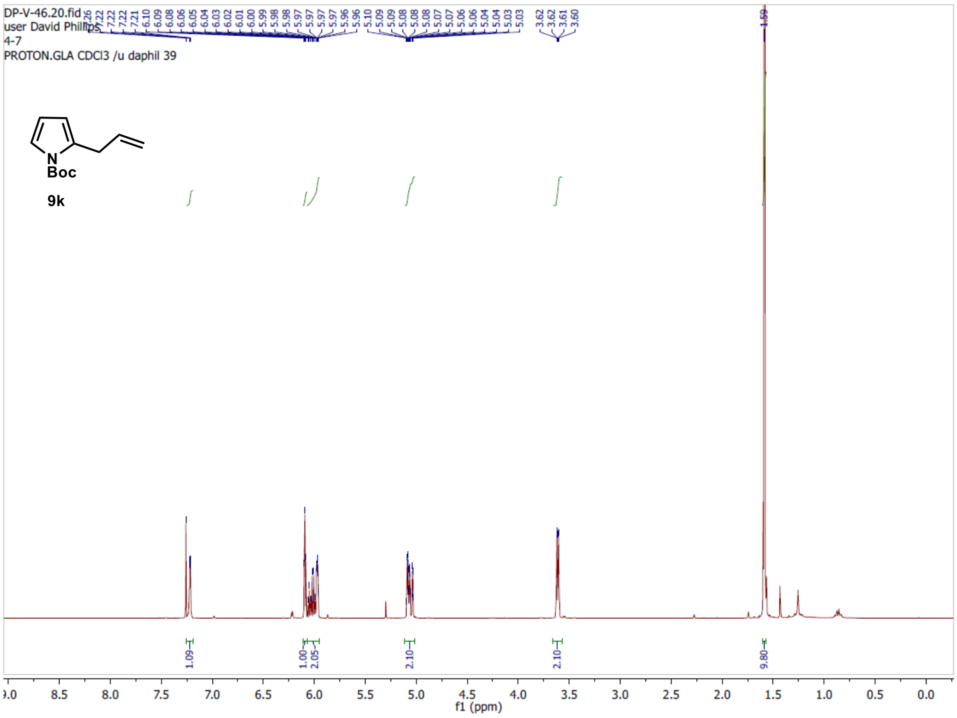
DP-V-46.20.fid
user David Phillips
4-7
PROTON.GLA CDCl3 /u daphil 39
N
Boc
9k
f1 (ppm)

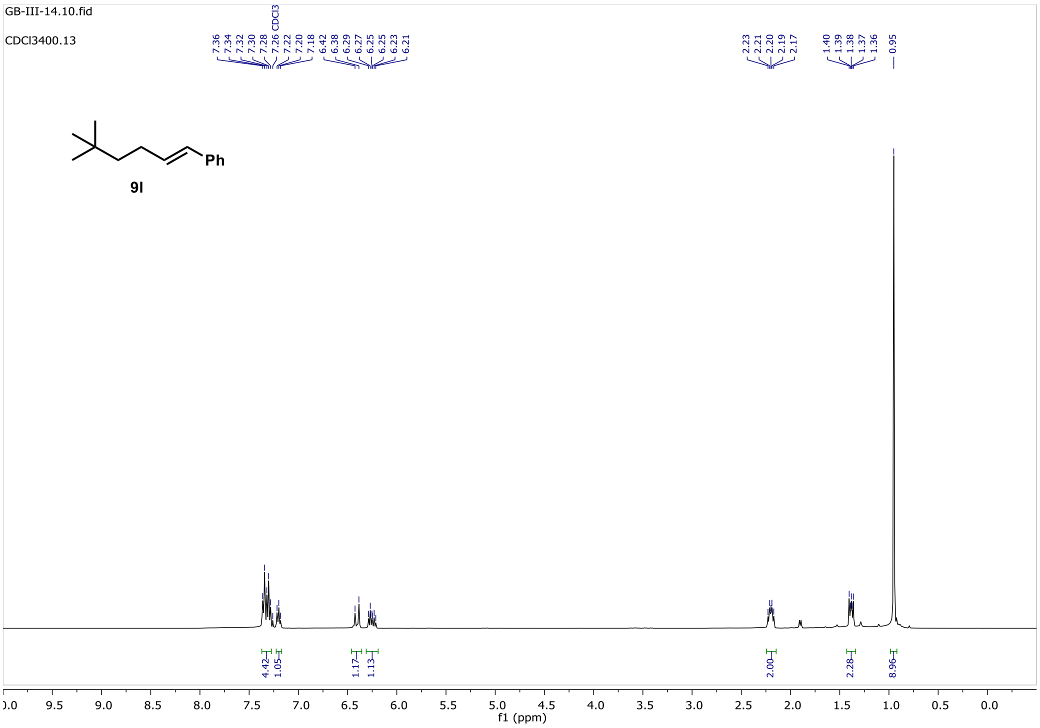
GB-III-14.10.fid
CDCl3400.13
Ph
9l
f1 (ppm)

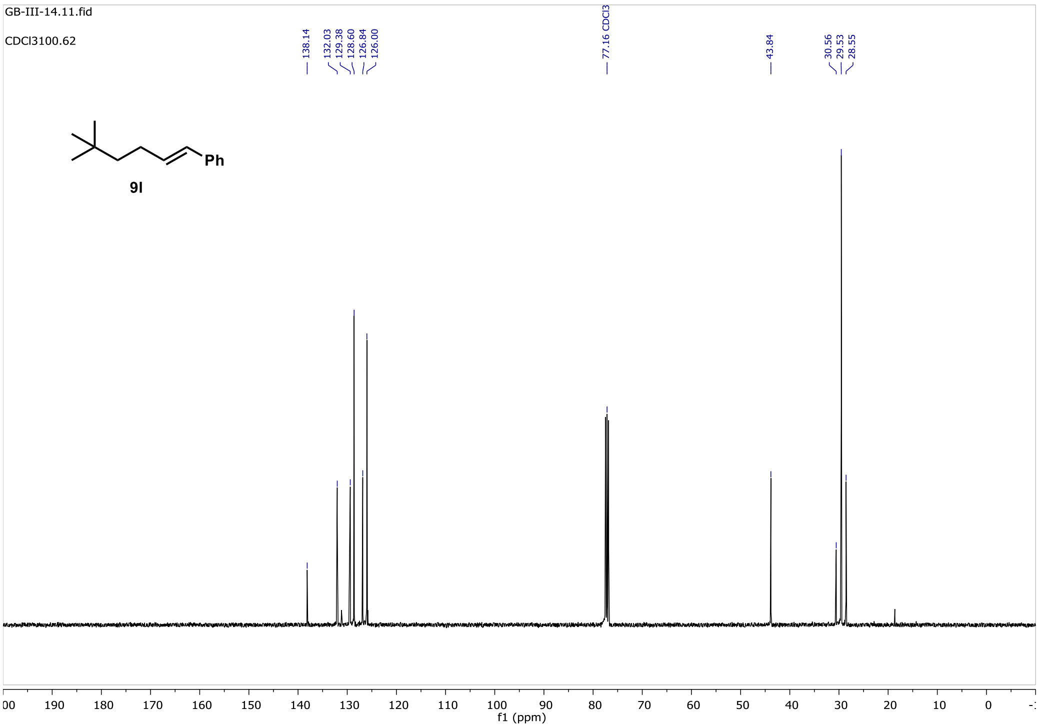
GB-III-14.11.fid
CDCl3100.62
138.14
132.03
129.38
128.60
126.84
126.00
77.16 CDCl3
43.84
30.56
29.53
28.55
Ph
9l
f1 (ppm)

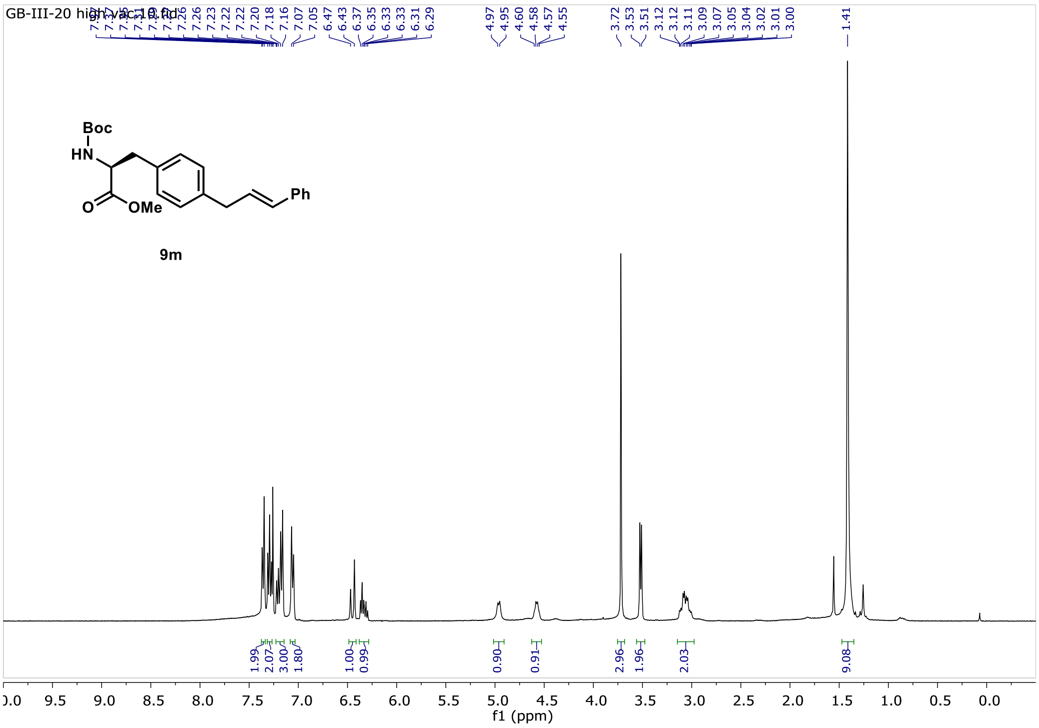
Boc
HN
O
OMe
Ph
9m
6.47
6.43
6.37
6.35
6.33
6.33
6.31
6.29
4.97
4.95
4.60
4.58
4.57
4.55
3.72
3.53
3.51
3.12
3.11
3.09
3.07
3.05
3.04
3.02
3.01
3.00
1.41
1.99
2.07
3.00
1.80
1.00
0.99
0.90
0.91
2.96
1.96
2.03
9.08
f1 (ppm)

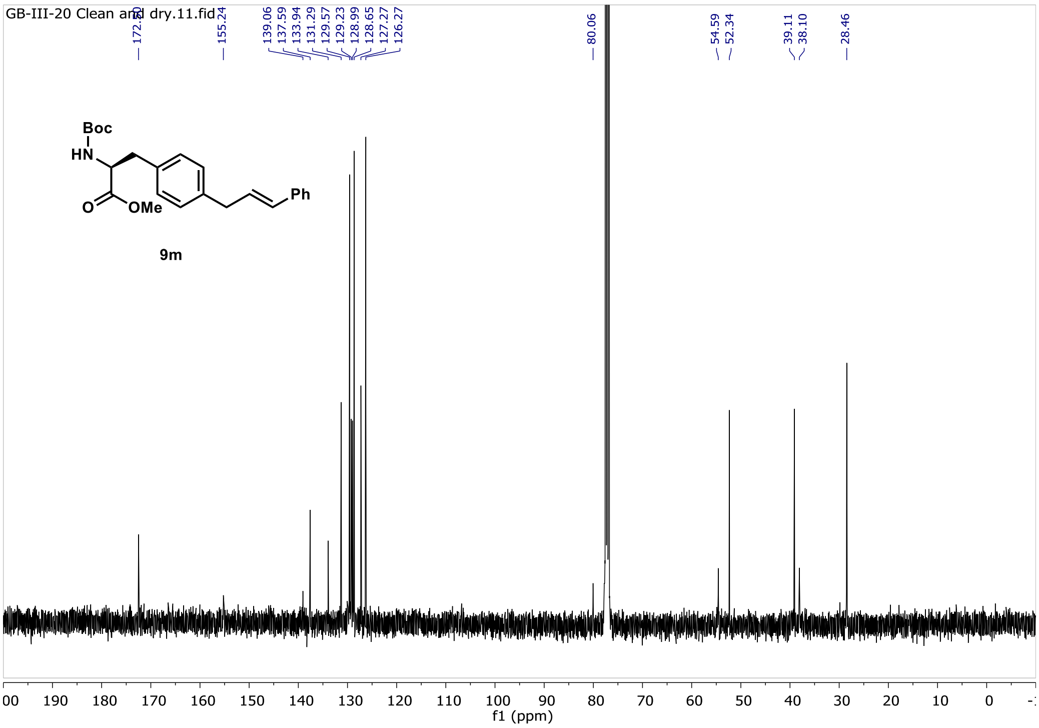
GB-III-20 Clean and dry.11.fid
172.20
155.24
139.06
137.59
133.94
131.29
129.57
129.23
128.99
128.65
127.27
126.27
80.06
54.59
52.34
39.11
38.10
28.46
Boc
HN
O
OMe
Ph
9m
f1 (ppm)

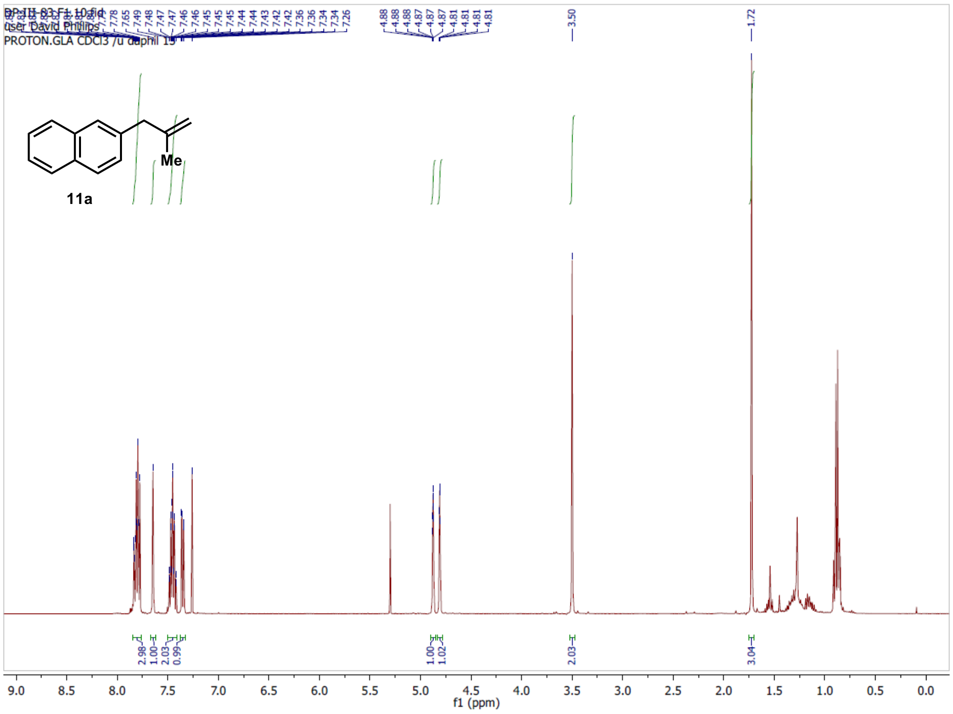
user David Phillips
PROTON.GLA CDCl3 /u dephil 15
3.50
1.72
Me
11a
2.98
1.00
2.03
0.99
1.00
1.02
2.03
3.04
f1 (ppm)

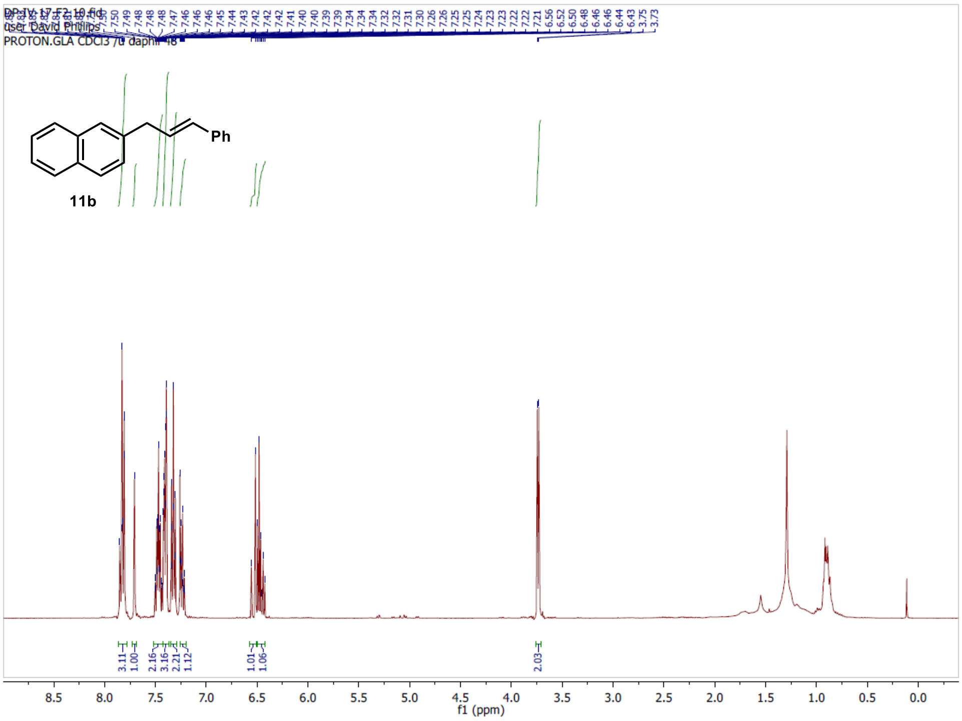
DP-IV-17-F2.10.fid
user David Phillips
PROTON.GLA CDCl3 /u daphil 18
Ph
11b
f1 (ppm)

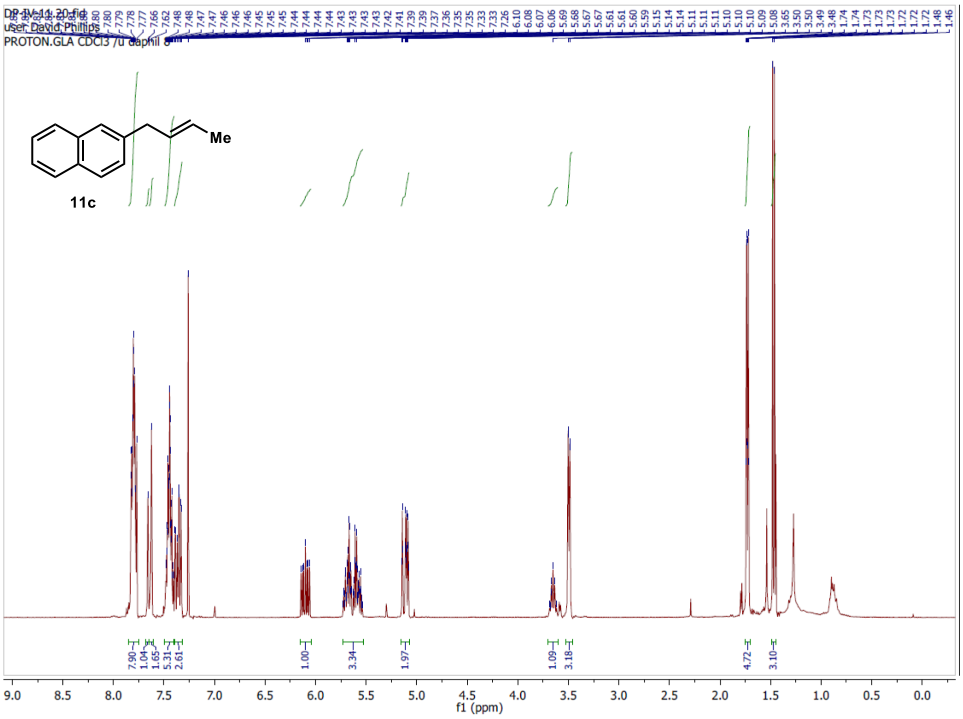
DP-IV-11.20.fid
user David Phillips
PROTON.GLA CDCl3 /u daphil 8
Me
11c
f1 (ppm)

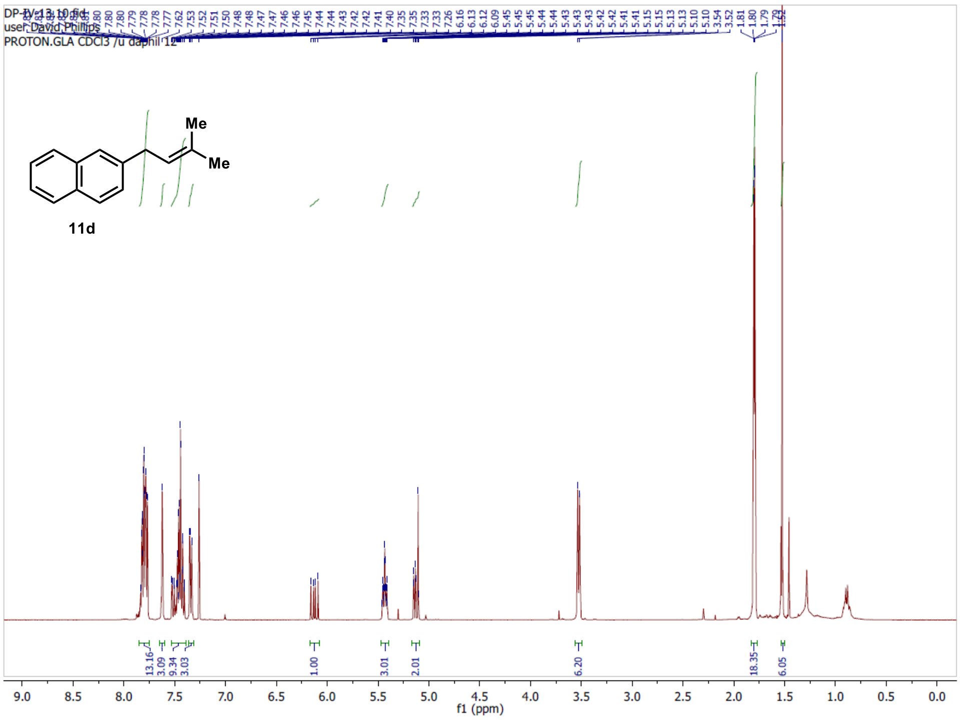
DP-IV-13.10.fid
user: David Phillips
PROTON.GLA CDCl3 /u daphil 12
Me
Me
11d
f1 (ppm)

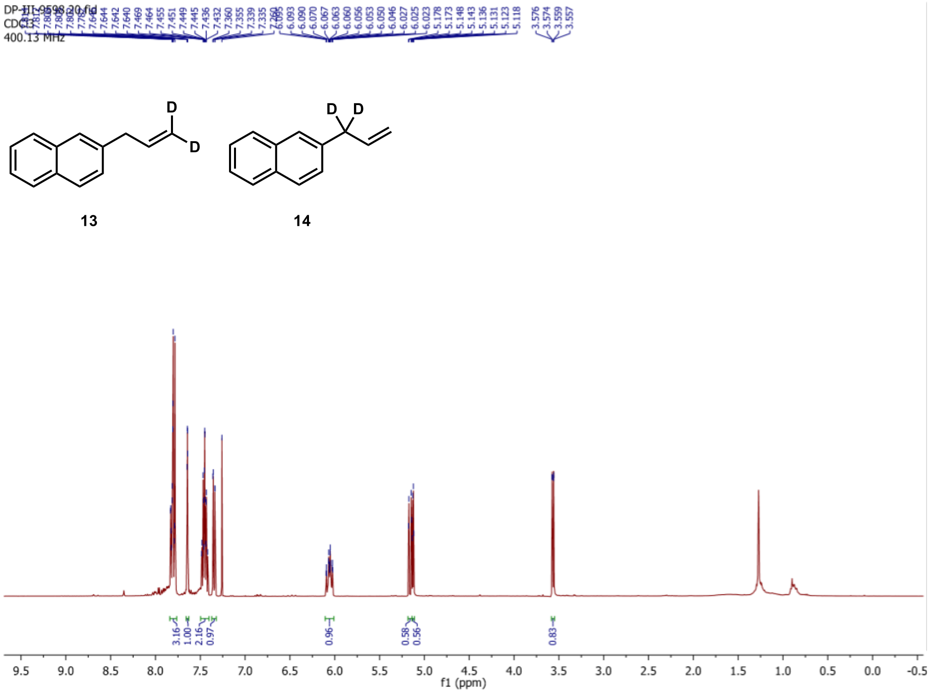
CDCl3
400.13 MHz
D
D
D D
13
14
f1 (ppm)

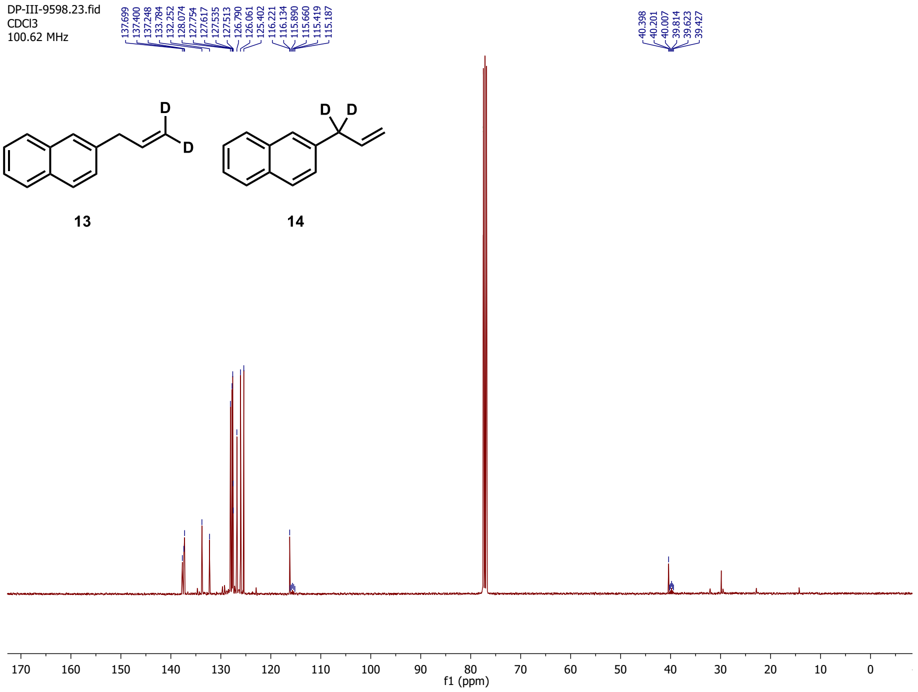
DP-III-9598.23.fid
CDCl3
100.62 MHz
137.699
137.400
137.248
133.784
132.252
128.074
127.794
127.617
127.535
127.513
126.790
126.061
125.402
116.221
116.134
115.890
115.660
115.419
115.187
40.398
40.201
40.007
39.814
39.623
39.427
D
D
D D
13
14
170 160 150 140 130 120 110 100 90 80 70 60 50 40 30 20 10 0
f1 (ppm)

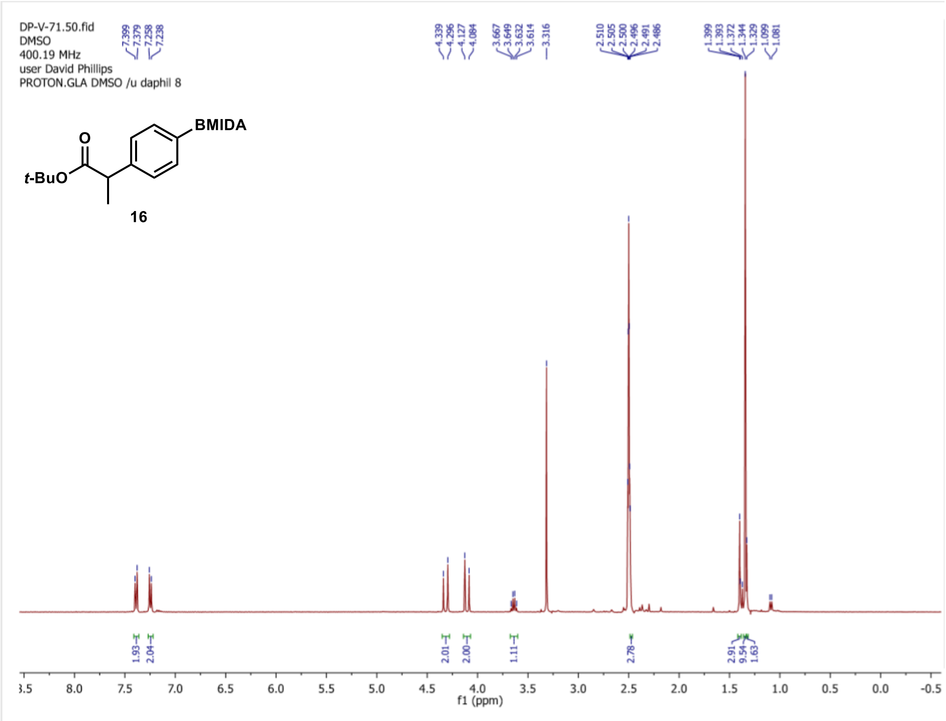
DP-V-71.50.fid
DMSO
400.19 MHz
user David Phillips
PROTON.GLA DMSO /u daphil 8
7.399
7.379
7.258
7.238
4.339
4.296
4.127
4.084
3.667
3.649
3.632
3.614
3.316
2.510
2.505
2.500
2.496
2.491
2.486
1.399
1.393
1.372
1.344
1.329
1.099
1.081
BMIDA
O
t-BuO
16
1.93
2.04
2.01
2.00
1.11
2.78
2.91
9.54
1.63
8.0 7.5 7.0 6.5 6.0 5.5 5.0 4.5 4.0 3.5 3.0 2.5 2.0 1.5 1.0 0.5 0.0 -0.5
f1 (ppm)

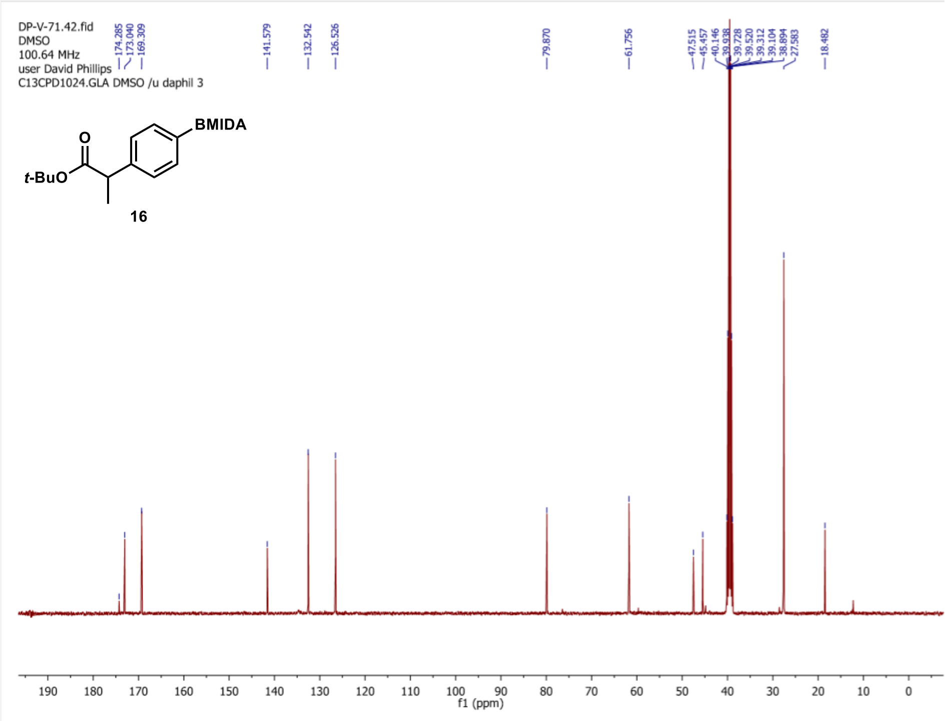
DP-V-71.42.fid
DMSO
100.64 MHz
user David Phillips
C13CPD1024.GLA DMSO /u daphil 3
174.285
173.040
169.309
141.579
132.542
126.526
79.870
61.756
47.515
45.457
40.146
39.938
39.728
39.520
39.312
39.104
38.894
27.583
18.482
BMIDA
O
t-BuO
16
f1 (ppm)

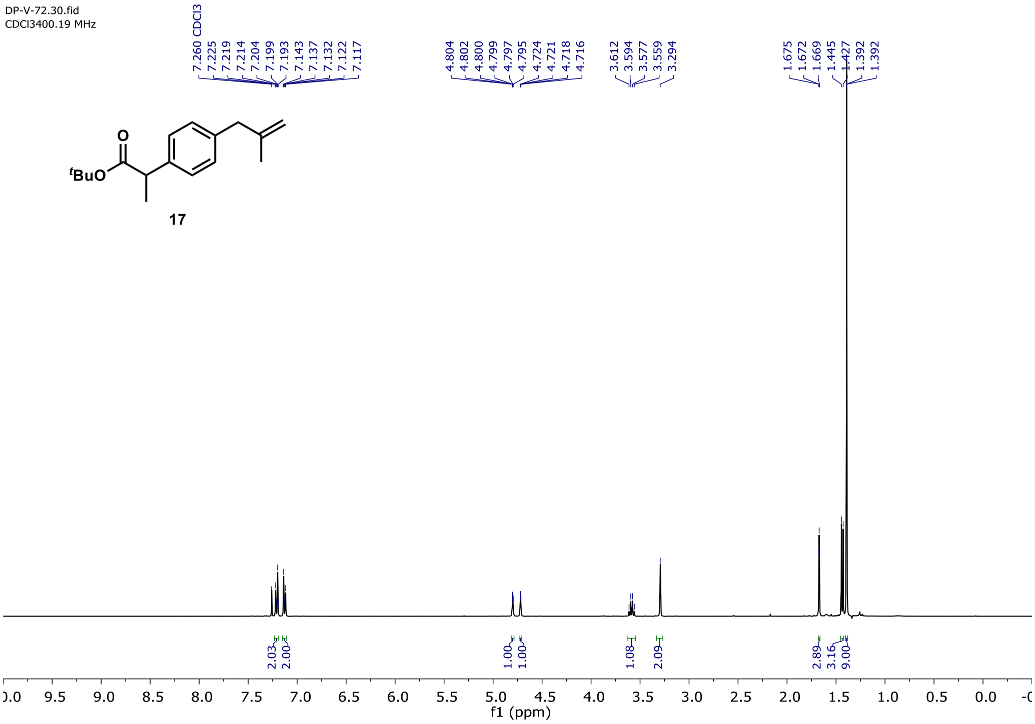
DP-V-72.30.fid
CDCl3400.19 MHz
7.260 CDCl3
7.225
7.219
7.214
7.204
7.199
7.193
7.143
7.137
7.132
7.122
7.117
4.804
4.802
4.800
4.799
4.797
4.795
4.724
4.721
4.718
4.716
3.612
3.594
3.577
3.559
3.294
1.675
1.672
1.669
1.445
1.427
1.392
1.392
O
tBuO
17
2.03
2.00
1.00
1.00
1.08
2.09
2.89
3.16
9.00
f1 (ppm)

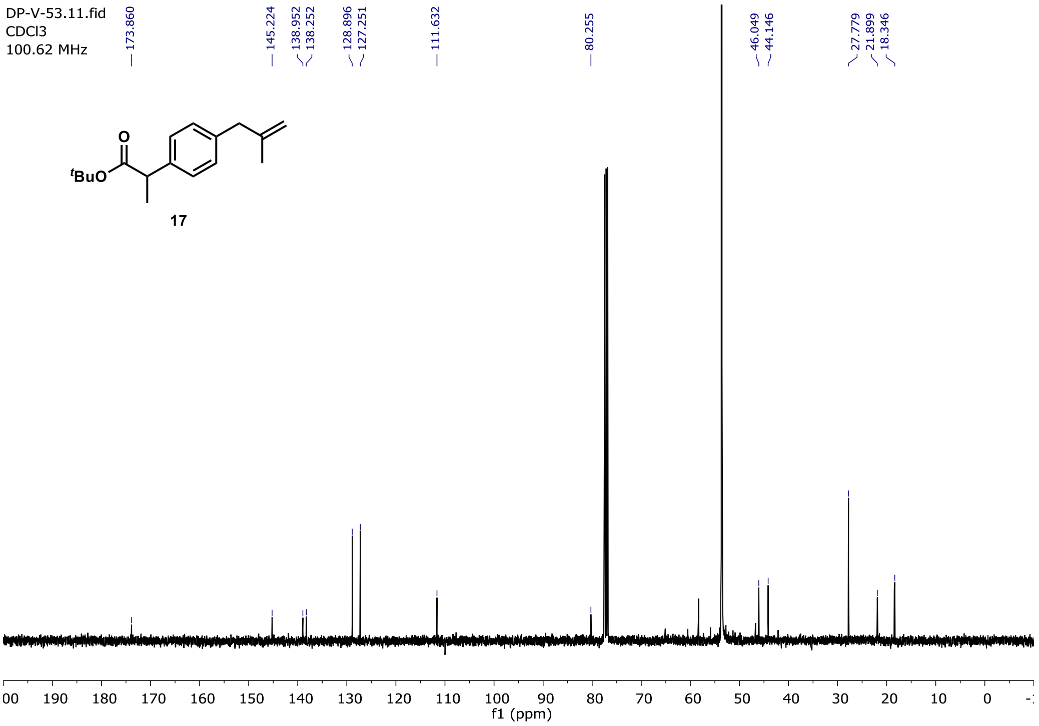
DP-V-53.11.fid
CDCl3
100.62 MHz
173.860
145.224
138.952
138.252
128.896
127.251
111.632
80.255
46.049
44.146
27.779
21.899
18.346
O
tBuO
f1 (ppm)

17

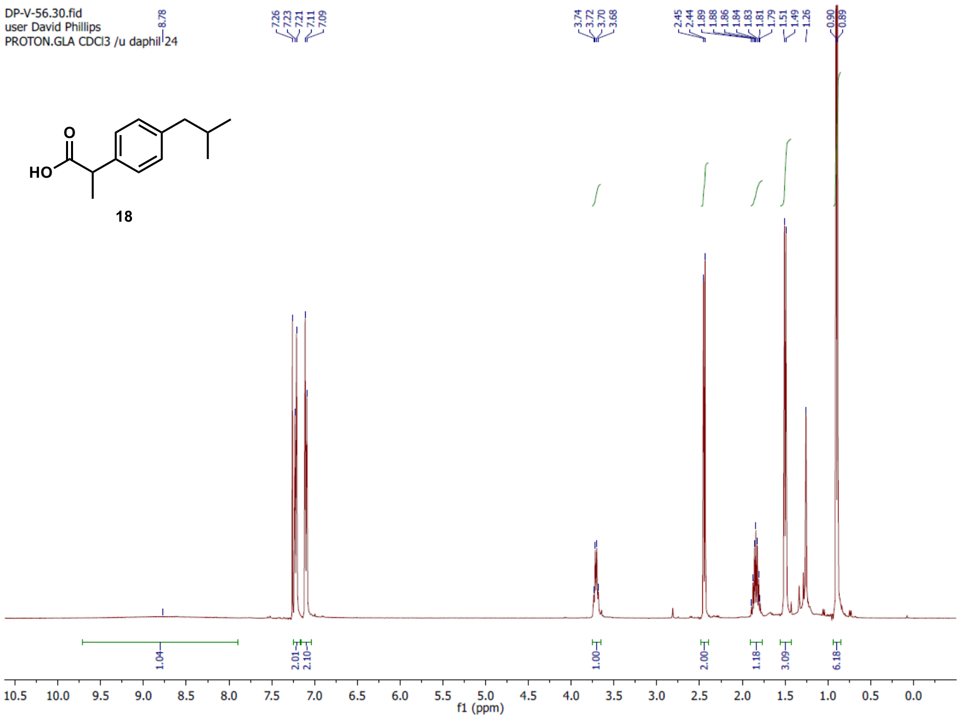
DP-V-56.30.fid
user David Phillips
PROTON.GLA CDCl3 /u daphil 24
8.78
7.26
7.23
7.21
7.11
7.09
3.74
3.72
3.70
3.68
2.45
2.44
1.89
1.88
1.86
1.84
1.83
1.81
1.79
1.51
1.49
1.26
0.90
0.89
O
HO
1.04
2.01
2.10
1.00
2.00
1.18
3.09
6.18
f1 (ppm)

18
